# Macrophages release IL11-containing filopodial tip vesicles and contribute to renal interstitial inflammation

**DOI:** 10.1186/s12964-023-01327-6

**Published:** 2023-10-18

**Authors:** Xiaodong Zhu, Yu Zhao, Yuqiu Liu, Wen Shi, Junlan Yang, Zhihong Liu, Xiaoliang Zhang

**Affiliations:** 1 Institute of Nephrology, Zhong Da Hospital, Southeast University School of Medicine, Nanjing, Jiangsu China; 2 Jinling Hospital, National Clinical Research Center of Kidney Diseases, Nanjing University School of Medicine, Nanjing, Jiangsu China

**Keywords:** Macrophage, Filopodial tip vesicle, IL11, Intercellular communication, Renal interstitial fibrosis, Tissue inflammation

## Abstract

Macrophage filopodia, which are dynamic nanotube-like protrusions, have mainly been studied in the context of pathogen clearance. The mechanisms by which they facilitate intercellular communication and mediate tissue inflammation remain poorly understood. Here, we show that macrophage filopodia produce a unique membrane structure called “filopodial tip vesicle” (FTV) that originate from the tip of macrophages filopodia. Filopodia tip-derived particles contain numerous internal-vesicles and function as cargo storage depots via nanotubular transport. Functional studies indicate that the shedding of FTV from filopodia tip allows the delivery of many molecular signalling molecules to fibroblasts. We observed that FTV derived from M1 macrophages and high glucose (HG)-stimulated macrophages (HG/M1-ftv) exhibit an enrichment of the chemokine IL11, which is critical for fibroblast transdifferentiation. HG/M1-ftv induce renal interstitial fibrosis in diabetic mice, while FTV inhibition or targeting FTV ^*IL11-*^ alleviates renal interstitial fibrosis, suggesting that the HG/M1-ftv^*IL11*^ pathway may be a novel mechanism underlying renal fibrosis in diabetic nephropathy. Collectively, FTV release could represent a novel function by which filopodia contribute to cell biological processes, and FTV is potentially associated with macrophage filopodia-related fibrotic diseases.

Video Abstract

**Supplementary Information:**

The online version contains supplementary material available at 10.1186/s12964-023-01327-6.

## Introduction

Filopodia are thin, dynamic actin-based protrusive structures that function as antennae through which cells probe their environment [1]. In recent years, some specialized filopodia, such as cytonemes, tunnelling nanotubes (TNTs), neutrophil trails and migrasomes, have been observed in diverse tissues and are capable of promoting signalling between specific cells over varying distances [2–4]. These protrusive structures may mediate signalling communication by delivering cargos such as mitochondria, endosomal vesicles and pathway ligands, in a highly specific manner [5]. Recently, this form of communication has been observed in macrophage filopodia, which are highly specialized cell protrusions that are primarily used to catch and retract pathogens [6]. For example, the long thin filopodia of mouse peritoneal macrophages and RAW 264.7 cell macrophages release 20–100 nm vesicular cholesterol-rich particles, suggesting their ability to deliver cell signalling molecules and mediate intercellular communication [7, 8]. However, less is known about how macrophage filopodia elicit signalling cascades or interact with neighbor cells in their environment.

Macrophages are key factors in the initiation and propagation of renal inflammation in diabetic nephropathy (DN) [9]. Once recruited to the kidney under pathological conditions, macrophages promote transdifferentiation of renal fibrosis into myofibroblasts, which is a crucial event in restoring homeostasis following renal injury [10, 11]. Targeting macrophage signalling pathways, by depleting tissue macrophages and inhibiting macrophage recruitment, has emerged as a potential strategy to attenuate the progression of renal fibrosis [12–14]. Although much has been learned about the complex interactions between macrophages and renal fibroblasts, the role of macrophage filopodia in the fibrotic process remains poorly understood.

Classically, in cell biology, macrophages activate renal fibroblasts through direct interaction and/or by secreting soluble molecules, such as hormones, growth factors and cytokines [15, 16]. Here, we document a highly specific manner in which macrophages exert their effects on renal fibroblasts via a unique type of membrane structure that is derived from the filopodia tip. Filopodia tip vesicles (abbreviated as “FTV”) fall into extracellular vesicles (EVs) category and allow molecular signals to be transmitted from macrophages to target cells in a selective manner. This work shows that macrophage filopodia-derived HG/M1-ftv^*IL11*^ signal transfer can be crucial to renal fibrosis in human and experimental models of kidney disease.

## Materials and methods

### Animal experiments

The experimental protocol for animal experimentation was approved by the ethics review committee of Southeast University. To establish diabetic nephropathy, C57/BL6 male mice (8 weeks of age, 20 ~ 25 g of body weight) received daily intraperitoneal injections of streptozotocin (STZ,50 mg/kg, Sigma, USA) for 5 consecutive days as previously reported [17]. To investigate the impact of FTV on renal interstitial fibrosis, recipient mice were administered M0-ftv, M1-ftv, M2-ftv, HG-ftv, M1-ftv^*IL11-*^, and HG-ftv^*IL11-*^ starting at week 8. Subsequently, the mice were euthanized at 12, 14, and 16 weeks of age. The intraparenchymal infusion was administered as previously described [18]. Briefly, animals were anesthetized, and the left kidney was exposed via flank incision. A solution containing approximately 50 ~ 100 × 10^6^ FTV or PBS was then injected into the left kidney via a 30-gauge needle into one to four sites, not exceeding a 100 µl volume. Sham mice underwent the same surgical procedures but with PBS injection. To address whether FTV are involved in renal interstitial fibrosis in DN, A group of STZ-treated mice was treated with the FTV inhibitor CK636 (100 µL,100 µM, TargetMol, USA) or Dynasore (100 µL, 80 µM, TargetMol, USA) through tail vein injections. The treatment began at week 12 and administered once a week for four weeks. Kidneys were harvested at 12, 14 and 16 weeks for real-time PCR and histological examination.

### Patient samples

Renal-biopsy specimens from 20 patients with DN were provided by Zhongda Hospital, Nanjing, China. The pathological diagnosis was confirmed by light microscopy, immunofluorescence assays and electron microscopic observations. Control tissues were obtained from the unaffected portion of surgical nephrectomies. The experiment was approved by the Ethics Committee of Zhongda Hospital.

### Cell culture and treatment

A mice macrophage cell line (RAW264.7) was obtained from Shanghai Bogoo Biotechnology Company (Shanghai, China). Nonpolarized macrophages (M0) were polarized into M1 and M2 macrophages by stimulation with 100 U/mL IFN-𝛾 plus 100 ng/mL LPS (M1 differentiation) (Sigma, USA) or 20 ng/mL IL4 (M2 differentiation) (Perotech) for 24 h, respectively. To explore the effect of high glucose on macrophage activation and FTV production, RAW264.7 cells were stimulated with high glucose (25 mM) for 24 h. To generate M1-ftv^*IL11-*^ and HG-ftv^*IL11-*^, RAW264.7 cells were transfected with IL11siRNA and subjected to different treatment conditions as described above. To block the formation of FTV, cells were pretreated with actin network modulators CK636 (100 µM) and Dynasore (80 µM) for 6 h before imaging. Rat kidney interstitial fibroblasts (NRK-49 F) were obtained from American Type Culture Collection (Manassas, USA). The cells were maintained in DMEM/F12 culture medium supplemented with 10% fetal bovine serum in a 37 °C, 5% CO2 incubator. After the cells were grown to 60–70% confluence in complete medium supplemented with10% FBS for 16 h, the cells were washed twice with serum-free medium.

### Macrophage FTV preparation

Macrophage FTV were isolated using procedures from previous reports with some modifications [19]. Briefly, nonpolarized macrophages, M1, M2 differentiated macrophages and HG treated macrophages were initially washed by serum-free cell culture medium, followed by treatment with trypsin. Subsequently, a low-speed centrifugation at 1000 × g for 10 min, then at 2000 x g for 20 min to remove cells and cellular debris. The collected cell culture medium was then mixed with an extraction buffer (Lysosome Isolation Kit, Sigma-Aldrich, USA), These crude FTV fraction were subjected to 150 000× g for 4 h ultracentrifugation at a multi-step 2%, 5%, 8%, 10%, 12%, 15%, 19%, 25%, 30% Optiprep density gradient medium solutions. The purified FTV fraction were then carefully collected and named M0-ftv, M1-ftv, M2-ftv, HG-ftv respectively. The prepared samples are subjected to further analysis or stored at -80 °C for subsequent processing. FTV size distribution and quantification of vesicles was analyzed by Nanoparticle tracking analysis (NTA) .

### Ultrastructure observation

To visualize FTV, we used in situ observation with transmission electron microscopy (TEM) and scanning electron microscopy (SEM). For TEM, RAW264.7 cells were seeded on sterile coverslips, washed with PBS, fixed with 2.5% glutaraldehyde, and post-fixed with 1% osmium. Dehydration was done with acetones and ethanol. After embedding in Eponate 12 resin, coverslips were carefully separated from the bottom of the sample blocks.70-nm-thick ultrathin sections were sliced and stained with uranyl acetate and lead citrate. Following air drying, the specimens were examined and imaged using TEM (FEI Tecnai G2 Spirit, USA). For the purified FTV and renal tissues, the sample processing follows the previously described TEM protocol [20]. For SEM, the cultured cells were cultivated on coverslips, fixed with 2.5% glutaraldehyde for 2 h. Subsequently, fixation was carried out with 1% osmium tetroxide for 1 h at room temperature. After undergoing dehydration through a graded ethanol series, the specimens were subjected to freeze drying. The dried samples were prepared for gold coating and examined by SEM (Hitachi S-4800, Japan).

### FTV immunogold labeling

FTV samples were fixed in 2% paraformaldehyde/0.02% glutaraldehyde for 2 h at room temperature. They were dehydrated with a gradient ethanol series (30%, 50%, 70%, 90%, 100%) and embedded in LR White Resin (Ted Pella, USA) at low temperature. Ultrathin Sect. (70 nm thickness) on nickel formvar grids were prepared. Grids were PBS-washed and blocked with 5% BSA-c (Aurion regents, Netherlands), then incubated with primary antibodies against Tetraspanin 4 (1:200 dilution, ab181995, Abcam) or integrin α5 (1:400 dilution, ab150361, Abcam). After washing, grids were exposed to secondary antibodies (10 nm goat anti-rabbit IgG gold, 1:25 dilution, Aurion regents, Netherlands) for 2 h at room temperature. Grids were stained with 2% uranyl acetate and examined using TEM (FEI Tecnai G2 Spirit).

### FTV uptake and functional studies

For FTV tracking, the purified FTV fractions (M0-ftv, M1-ftv, M2-ftv, HG-ftv, HG/M1-ftv^*IL11-*^) were labelled with the red lipophilic fluorescent dye PKH26 (PKH26 fluorescent cell linker kit, Sigma‒Aldrich) according to the manufacturer’s instructions. To determine the effect of FTV, fibroblasts (NRK-49 F) were cultured on 35 mm dish and co-cultured with PKH26-labelled FTV for 24 h at 37 °C. Then the cells were incubated with primary antibodies (rabbit polyclonal α-SMA antibody, 1:100 dilution, Abcam; rabbit polyclonal collagen I antibody, 1:100 dilution, Abcam) overnight at 4 °C, followed by incubation with secondary antibodies (goat anti-rabbit IgG H&L Alexa Fluor® 488,1:1000 dilution, Abcam) for 2 h at 37 °C. Nuclei were stained with DAPI (Beyotime Biotechnology, Shanghai, China). Confocal microscopy (Leica, Germany) was used to examine the stained fibroblasts.

To examine the effects of IL11 on fibroblasts, fibroblasts were incubated with 5 ng/ml recombinant mouse IL11(Z200125, Cell Biologics Inc) for 24 h, then levels of the fibroblasts markers α-SMA and collagen I were examined as described above. To block the IL11 signalling pathway ,fibroblasts were incubated with 2 µg/ml anti-IL11 receptor antibody (sc-130,920, Santa Cruz ) or an IgG control antibody for 1 h, and then co-cultured with PKH26-labelled M1-ftv or HG-ftv for 24 h. Then the fibroblasts α-SMA and collagen I immunostaining were performed as described above. MTT (Sigma, USA) assays was used to detect fibroblasts proliferation.

### Immunohistochemistry and histology assay

To evaluate kidney histological changes and interstitial fibrosis, we performed immunohistochemistry on paraffin sections. The sections were deparaffinized, rehydrated, and stained using the EnVision system protocol (DAKO Corp, USA). After blocking with 5% BSA-c, the sections were incubated overnight with an anti-α-SMA antibody (1:100 dilution) and anti-collagen I antibody (1:100 dilution) at 4 °C overnight and visualized using a horseradish peroxidase-conjugated goat anti-rabbit antibody and DAB. As negative controls, all the specimens were incubated with an isotype-matched control antibody. Image-Pro-Plus image analysis software was used to calculate the number of positive cells and area of positive staining, and the means were determined. All the measurements and scorings were performed in a blinded manner. For histology analysis, paraffin-embedded kidney sections were stained with H&E and Masson’s trichrome by standard procedures. The fibrotic lesion areas were quantified in 10 randomly nonoverlapping fields under 400× magnification and expressed as a percentage of the total field area.

### Transfection and RNA interference

The recombinant Tetraspanin 4-GFP vectors (Vector Builder Inc., China) were transfected into RAW264.7 cells with Lipofectamine 2000 (Invitrogen, USA). After screening with G418, a stably transfected cell line was established, and the transcription and expression of Tetraspanin 4 were measured by RT‒PCR, Western blotting and immunofluorescence. Transfection of siRNA was carried out according to Santa Cruz’s protocol. Briefly, RAW264.7 cells were seeded in 6-well plates (1 × 10^5^ cells/well) and transfected with 10 nM IL11 siRNAs (sc-39,637, Santa Cruz) in transfection medium containing Lipofectamine 2000 (Invitrogen, USA). The cells were transfected for 6 h at 37 °C and subsequently cultured in normal medium overnight before being subjected to different treatment conditions.

### Genome-wide expression profiling of FTV

To characterize the FTV gene expression signatures that might underlie the FTV-associated profibrotic effect, M0-ftv, M1-ftv, M2-ftv and HG-ftv genome-wide expression profiling was carried out by using SurePrint G3 Mouse GE V2.0 8 × 60 K Microarrays (Design ID: 074809, Agilent Technologies, USA). Total RNA was quantified by a NanoDrop ND-2000 (Thermo Scientific), and RNA integrity was assessed using an Agilent Bioanalyzer 2100 (Agilent Technologies). Sample labelling, microarray hybridization and washing were performed based on the manufacturer’s standard protocols. Briefly, total RNA was transcribed into double-stranded cDNA, synthesized into cRNA and labelled with cyanine-3-CTP. The labelled cRNAs were hybridized onto the microarray. After washing, the arrays were scanned by an Agilent Scanner G2505C (Agilent Technologies). For data analysis, Feature Extraction software (version 10.7.1.1, Agilent Technologies) was used to analyse array images to obtain raw data. Genespring (version 14.9, Agilent Technologies) was used to complete the basic analysis with the raw data. First, the raw data were normalized with the quantile algorithm. Differentially expressed genes were then identified according to fold change in expression. Up- and downregulated genes were identified according to a fold change in expression > = 2.0. Afterwards, GO analysis and KEGG analysis were applied to determine the roles of these differentially expressed mRNAs.

### Statistical analyses

All data are shown as means ± SD as indicated. For experiments comparing 2 groups, the results were analyzed using the t test. When > 2 groups were compared, 1-way analysis of variance followed by Dunnett’s test was employed to analyze the differences using SPSS version 20.0. For all tests, *p* values < 0.05 were considered statistically significant.

## Results

### Macrophage filopodia release a unique vesicular membrane structure in vitro

**Fig. 1:**
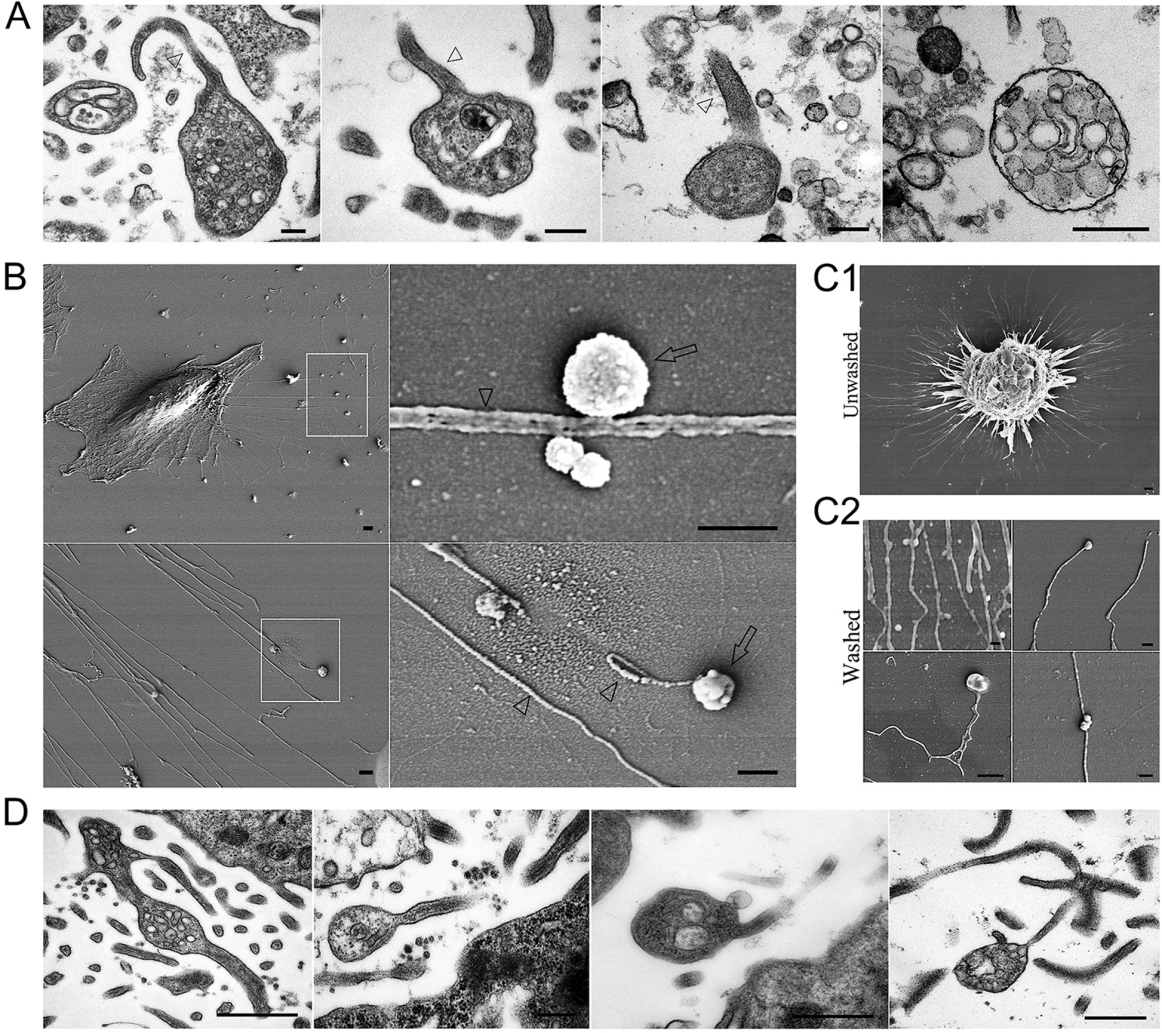
Representative images of FTV. **A** TEM images of FTV characterized by nanotubular filopodia (triangle). Scale bars: 200 nm. **B** SEM reveals that FTV(arrow) typically grow either on or at the tips of the nanotubular filopodia (triangle). Scale bars: 500 nm. **C1** SEM images of macrophage cells (RAW264.7) on the coverslip surface. **C2** Some residual FTV on the coverslip surface after extensive washing. Scale bar: 500 nm. **D** In situ TEM images of small internal vesicles were observed within the nanotubular filopodia as well as in the tip vesicle of FTV. Scale bar: 500 nm

The secretion of all kinds of extracellular vesicles (EVs) involves highly conserved manner exploited by diverse cells as a mode of intercellular communication [21]. When a thorough investigation of nanoparticles in culture media from macrophages(RAW264.7) was performed with the aim of identifying EVs, some vesicle-like structures whose surfaces were connected by long nanotubular filopodia were identified, by transmission electron microscopy (TEM), as shown in Fig. 1A. These unique filopodia membrane structures of macrophages have not yet been reported. The attached nanotubules were approximately 80 ~ 120 nm in diameter, and the majority of vesicle structures were oval shaped, and comparable in size, ranging from 500 to 1500 nm. With high magnification, heterogeneous contents and numerous internal-vesicles were observed in these structures. Most internal-vesicles have their own membrane boundaries. The number of internal-vesicles varied greatly, and the size was estimated to be 40 ~ 125 nm. Some structures formed bulb-like vesicles due to volume expansion. Overall, this unique filopodia membrane structure had characteristics of EVs, but were different from classical EVs, such as exosome, microvesicle and apoptotic bodies [22, 23].

To determine how these unique membrane structures were produced, we decided to characterize them in an in-depth study. RAW264.7 cells were seeded on cover slips and processed in situ. As revealed by scanning electron microscopy (SEM), abundant nanotubular filopodia extended from the cell body of macrophage. We confirmed that some topologically similar vesicular structures typically grew on the tips of nanotubular filopodia (Fig. 1B). The membrane structures ranged from 500 to 1500 nm in size, or even larger, and they were similar in size to the vesicular structures that were identified in the cell culture media. To rule out the possibility that the unique vesicle structures were generated by fusion or budding from macrophages, we next focused on macrophage-experienced assay glass coverslips (Fig. 1C1). After the cultured macrophage were completely removed by extensively washing the coverslip surface. Unexpectedly, we could not detect any remaining macrophages bodies on the glass surface, but we did observe a substantial amount of microvillous particles that had deposited on the coverslip (Fig. 1C2). According to SEM observation, these structures were later confirmed as the nanotubular filopodia and vesicular structures that we identified above. Until now, there has been limited literature documenting the production of vesicle structures by macrophage filopodia. To highlight the unique membrane structures generated from macrophage filopodia, we refer to them as "Filopodial Tip Vesicles," abbreviated as "FTV".

Why do macrophage filopodia produce the vesicular structures in this manner? FTV contain numerous intralumenal vesicles, so it is important to understand how these vesicles are formed, sorted and transported into FTV. In situ TEM provided an information about the internal structure of these FTV and additional details. As shown in (Fig. 1D), the nanotubular filopodial like thin membrane channel or bridge offers one route for transferring of cytoplasmic cargo into the attached vesicle compartment. Some cellular contents, such as internal vesicles and cytosol, are located in nanotubular filopodia. FTV like functions as a storage depot that aggregate with cytoplasm and numbers of small internal vesicles, suggesting that the FTV dynamically engage in transportation and cargo storage.

### FTV are identified in renal interstitium of DN patients and diabetic mice

**Fig. 2:**
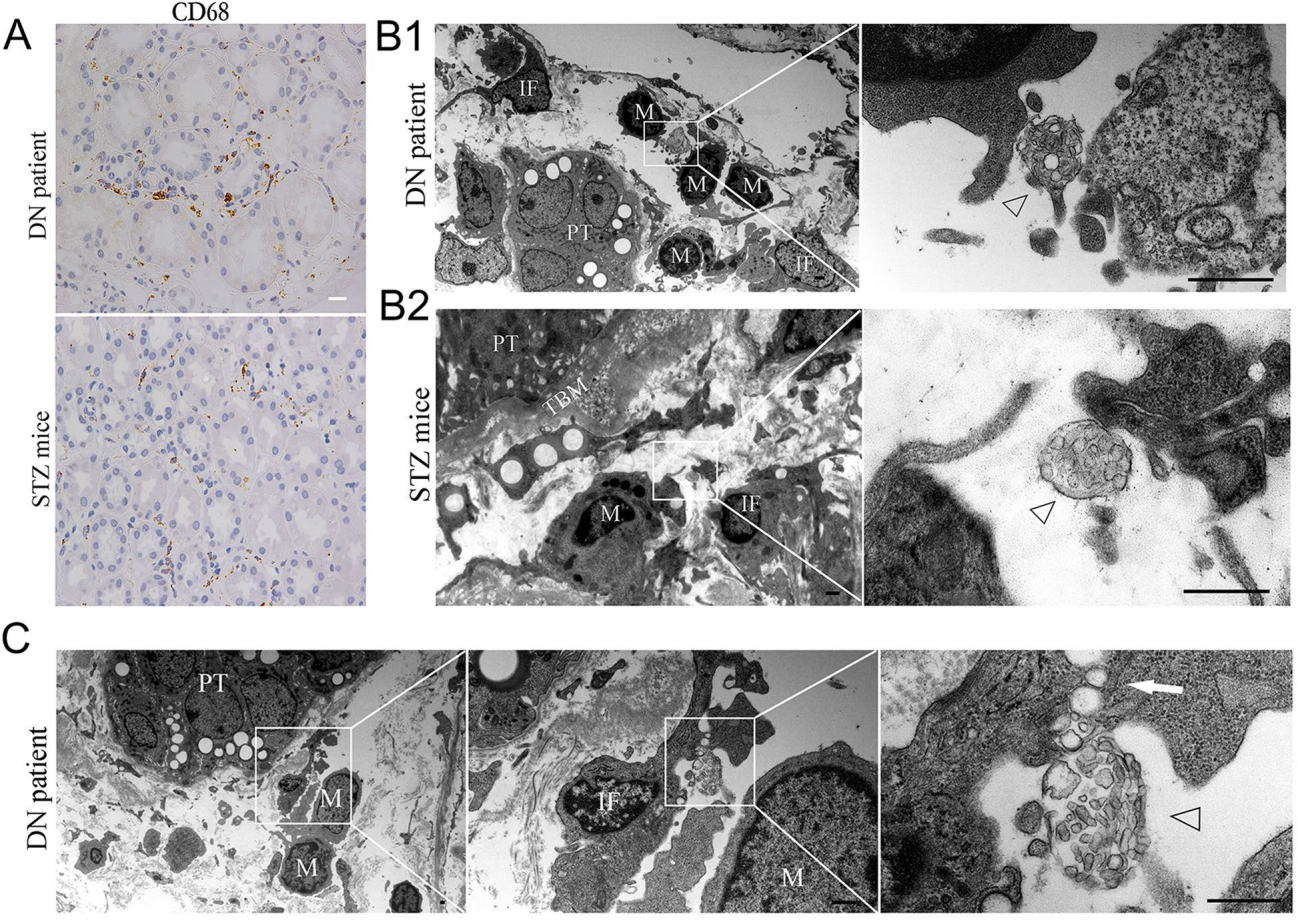
FTV are identified in renal interstitium of DN patients and diabetic mice. **A** Immunostaining images of CD68 macrophages in the renal interstitium of DN patients and STZ mice. Scale bar: 10 μm. **B1** TEM images of FTV-like nanoparticles (arrow) in the renal interstitium of DN patient, **B2** as well as in STZ mice. (M: macrophages, IF: fibroblasts). Scale bar: 1 μm. **C** TEM images of FTV interact with renal fibroblast. Progressive Zooms into the TEM image reveal macrophage (M) scattered in close to fibroblasts (IF), characterized by the collagen fibers. FTV (triangle) and its internal vesicles (arrow) appear to have possibly been internalized by renal fibroblasts in DN patients. (PT: proximal renal tubule, TBM: basement membrane of renal tubules). Scale bar: 500 nm

Macrophages are the major inflammatory cells involved in renal interstitial inflammation in DN [24], therefore, it is crucial to determine whether FTV are also present in vivo. The existence of FTV in both patients with DN and mice with streptozotocin-induced diabetic mice (STZ mice) model was examined. We found that macrophages accumulated in the renal interstitium of patients with DN and STZ model mice (Fig. 2A). Some FTV-like vesicles from these macrophages were observed. These FTV-like structures presented typical morphology via connecting-filopodia (Fig. 2B1), making them readily distinguishable from other types of EVs. Next, we conducted a search for the presence of FTV in the kidneys of mice with STZ mice. As expected, morphologically similar FTV vesicles were observed in the interstitial space of kidney from STZ-induced mice (Fig. 2B2).

The renal interstitium contains a large proportion of resident fibroblasts that are critical for tissue homeostasis and renal fibrosis in DN [25]. Given that FTV are nanoscale particles with the potential to traverse in the renal interstitial space, we investigated their potential interactions with the resident fibroblast. The mice model study indicated that some FTVs could be detected near fibroblast peripheries in renal interstitium. Notably, it appears that some FTV cargoes or intervesicles have potentially been internalized by fibroblasts through an endocytic pathway (Fig. 2C). This observation reveals that FTV probably serve as carries of signalls and play a potential role on renal fibroblast. In summary, we conclude that the release of FTV is a fundamental cell–cell communication pattern that is exploited by macrophages.

### FTV differ from classical EVs in biophysical and morphological properties

**Fig. 3:**
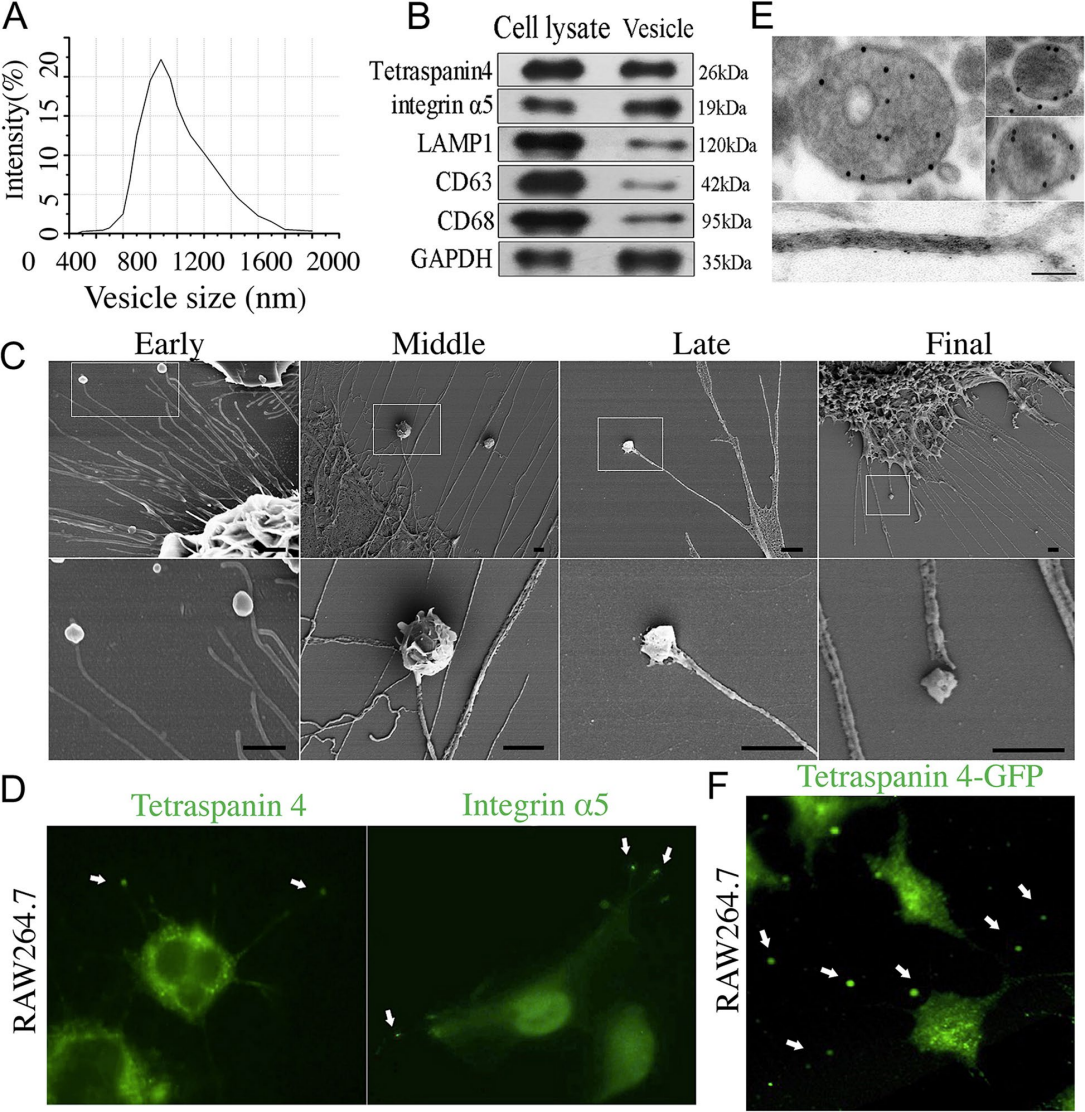
Characterization of FTV. **A** The particle size distribution histogram measured by NTA. **B** Western blot of Tetraspanin 4, integrin α5, LAMP1, CD63 and CD68 in FTV and cell lysate of macrophage. *n* = 5. **C** SEM images of FTV on different formation stages. In the initial stage, FTV originated from the primary filopodia. During the middle stage, the size of FTV increases while they maintain their connection to the filopodia. Following that, some FTV displayed surface cracks. Finally, FTV were released from the filopodia and scattered in the vicinity. Scale bar: 500 nm. **D** Immunofluorescence staining of Tetraspanin 4 (green) and Integrin α5 (green) in FTV. 40x objective. **E** Immunogold labeling of Tetraspanin 4 was detected in the plasmalemma of FTV (above) and in nanotubular filopodia (below). Scale bar: 250 nm. **F** Tetraspanin 4-GFP marked FTV (arrows). 40x objective

Next, we aimed to explore the biogenesis and general characteristics of FTV. FTV preparation were purified from the supernatant of cells culture medium, and size distribution analysis and quantification were performed using nanoparticle tracking analysis (NTA). The FTV cluster showed relative variation in scattering intensity, and the global mean was much bigger than that of exosomes and microvesicles in size (Fig. 3A). Although morphologically reminiscent of multivesicular body (MVBs), when the expression of LAMP1, a marker of mature/late endosomes and MVBs was examined in FTVs, its expression was found to be generally lower levels (Fig. 3B), which is consistent with earlier observations [26]. In addition, the expression of CD63, a marker of exosome, was nearly undetectable in FTV, but the expression of CD68 was detected. Some other vesicular structures such as oncosomes and spheresomes are comparable to FTV in size [27, 28], however, they are produced by budding and are completely different from FTV in genesis. To demonstrate how FTV formation, we propose that the formation of FTV consists of four stages (Fig. 3C). In general, FTV derived from the tip of nanotubular filopodia in the early stage of formation. In the middle period, FTV remained connected to the filopodia, but some FTV appear to have surface cracks. Following that, the internal contents are expelled from the cracedk FTV. In the final stage, FTV disconnect from the primary filopodia and the separated vesicles cargoes lie peripherally.

To further determine the relationship between FTV and migrasome, we tried to detect FTV in situ with an anti-Tetraspanin 4 antibody that labels migrasome. Immunofluorescence staining analysis revealed that Tetraspanin 4 was highly enriched in both FTV and the original nanotubular filopodia. Integrin α5, another currently available marker for migrasome, also specifically localizes on FTV, but is present at very low levels on its original nanotubular filopodia (Fig. 3D). Similar specific labelling results were obtained byTetraspanin 4 immunogold-labelling at the ultrastructural level (Fig. 3E). In this respect, FTV might share certain features and biomarkers with migrasome. In particular, we generated a stable Tetraspanin 4-GFP RAW264.7 cell line expressing Tetraspanin 4-GFP by transfection (pLV27-EGFP: T2A: Puro-EF1A > mTetraspanin 4) (Fig. 3S). As expected, Tetraspanin 4-GFP was highly enriched in FTV (Fig. 3F). Based on such findings, it would seem as if FTV share some properties of migrasome.

### Stimulations modulate the production of FTV

**Fig. 4:**
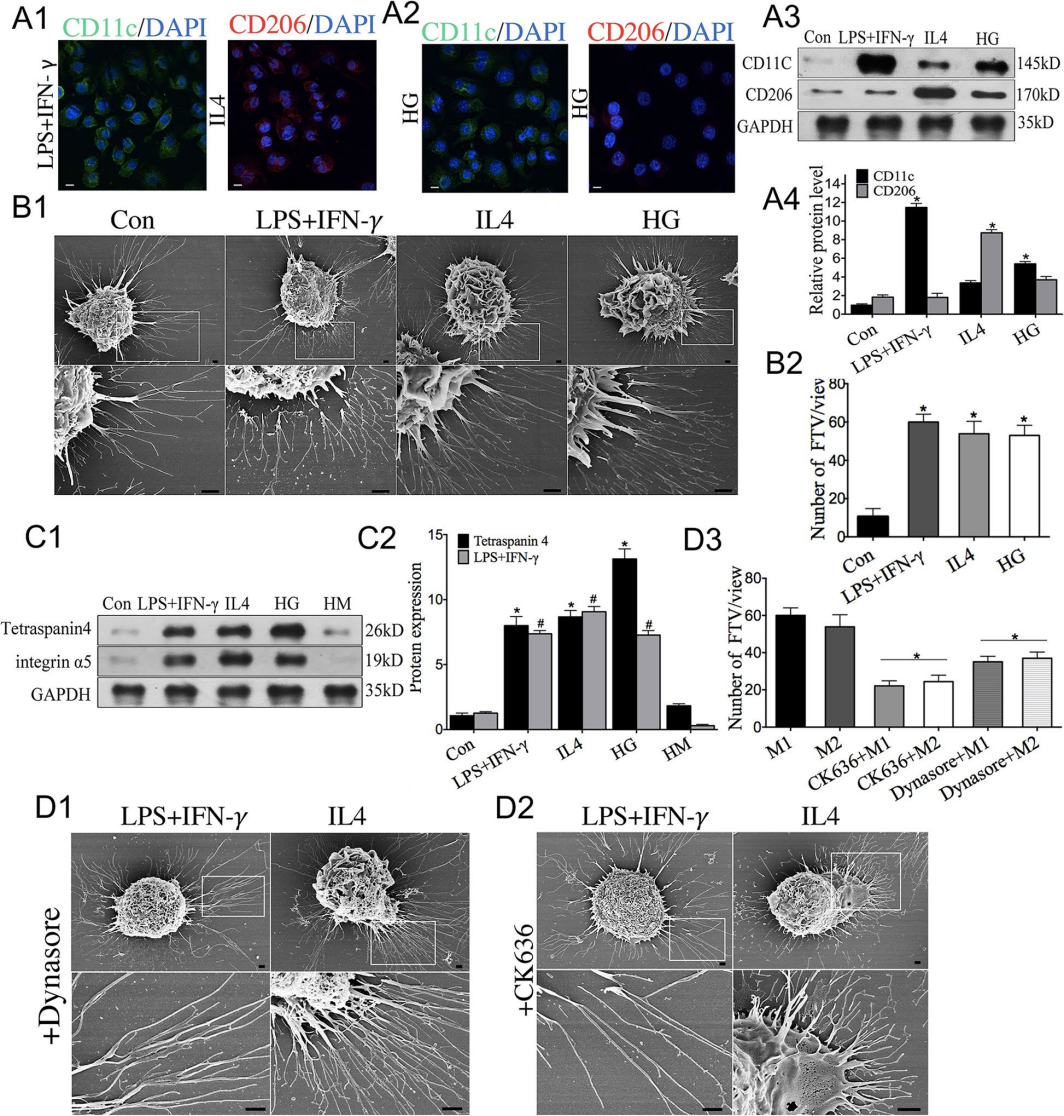
Stimulations modulate production of FTV. **A1**-**A2** Immunofluorescence images of activated macrophage cells stained with CD11c (green) and CD206 (red) under stimulations of IFN-γ plus LPS, IL4 and HG conditions. Nuclei were stained with DAPI (blue). Scale bar: 10 μm. **A3-A4** CD11c and CD206 levels revealed by Western blotting. *n* = 3. **B1**-**B2** SEM images of macrophages treated with LPS plus IFN-γ, IL4, and HG with a statistical analysis of FTV counts. Scale bar: 1 μm. *n* = 10. **C1-C2** The impact of LPS plus IFN-γ, IL4, and HG on macrophage cell Tetraspanin 4 and integrin α5 expression levels as revealed by Western blotting. high mannitol (HM; osmotic pressure control). *n* = 10. **D1**-**D2** SEM images show the impact of CK636 or Dynasore on production of FTV and the analysis of FTV counts. (M1:LPS plus IFN-γ, M2:IL4 treat). Scale bar: 1 μm. *n* = 10. *p < 0.05 *vs*. the control group, the M1 or M2 group and ^*#*^*P* < 0.05 *vs*. the control group

Given that the number of filopodia increased after macrophage activation [29], we stimulated nonpolarized macrophages (M0 phenotype) with endotoxin lipopolysaccharide (LPS) in combination with interferon-γ (IFN-γ) or IL4 to generate classically activated (M1 phenotype) macrophages or alternatively activated (M2 phenotype) macrophages. Immunofluorescence results demonstrate that macrophages stimulated with IFN-γ and LPS express CD11C, indicating M1 macrophages, while those stimulated with IL-4 express CD206 (Fig. 4A1). Macrophages stimulated with HG exhibited higher levels of CD11c and lower expression of CD206 according to immunofluorescence staining and flow cytometric analysis (Fig. 4A2, 4 S), Western blotting analysis confirmed that macrophages treated with HG significantly exhibited increased expression of CD11c (Fig. 4A3-A4) suggesting that macrophages undergo M1 polarization when exposed to HG stress. Next, the effect of M1and M2 macrophage on FTV production was evaluated by SEM, the results showed a significant increase in the abundance of FTV both in M1 and M2 macrophages (Fig. 4B1-B2). Furthermore, high glucose (HG) treat macrophages led to marked filopodia formation, accompanied by a notable increase in FTV production compared to the control group.The Western blotting results further revealed a significant increase in the expression levels of Tetraspanin4 and integrin α5 both in M1 and M2 macrophages 4 (Fig. 4C1-C2). The results above suggest that activated macrophages promote filopodia formation and an increased production of FTV.

To observe whether the blocking filopodia can reduce the formation of FTV, RAW264.7 cells were pretreated with the filopodia inhibitor CK636 or Dynasore. SEM revealed that the effect of LPS, IFN-γ or IL4 on FTV production was significantly attenuated and decreased to control levels (Fig. 4D1-D3). These results reveal that HG affects macrophage activation, and activated macrophages exhibit increased FTV production.

### Fibroblast activation is associated with M1-ftv and HG-ftv

**Fig. 5:**
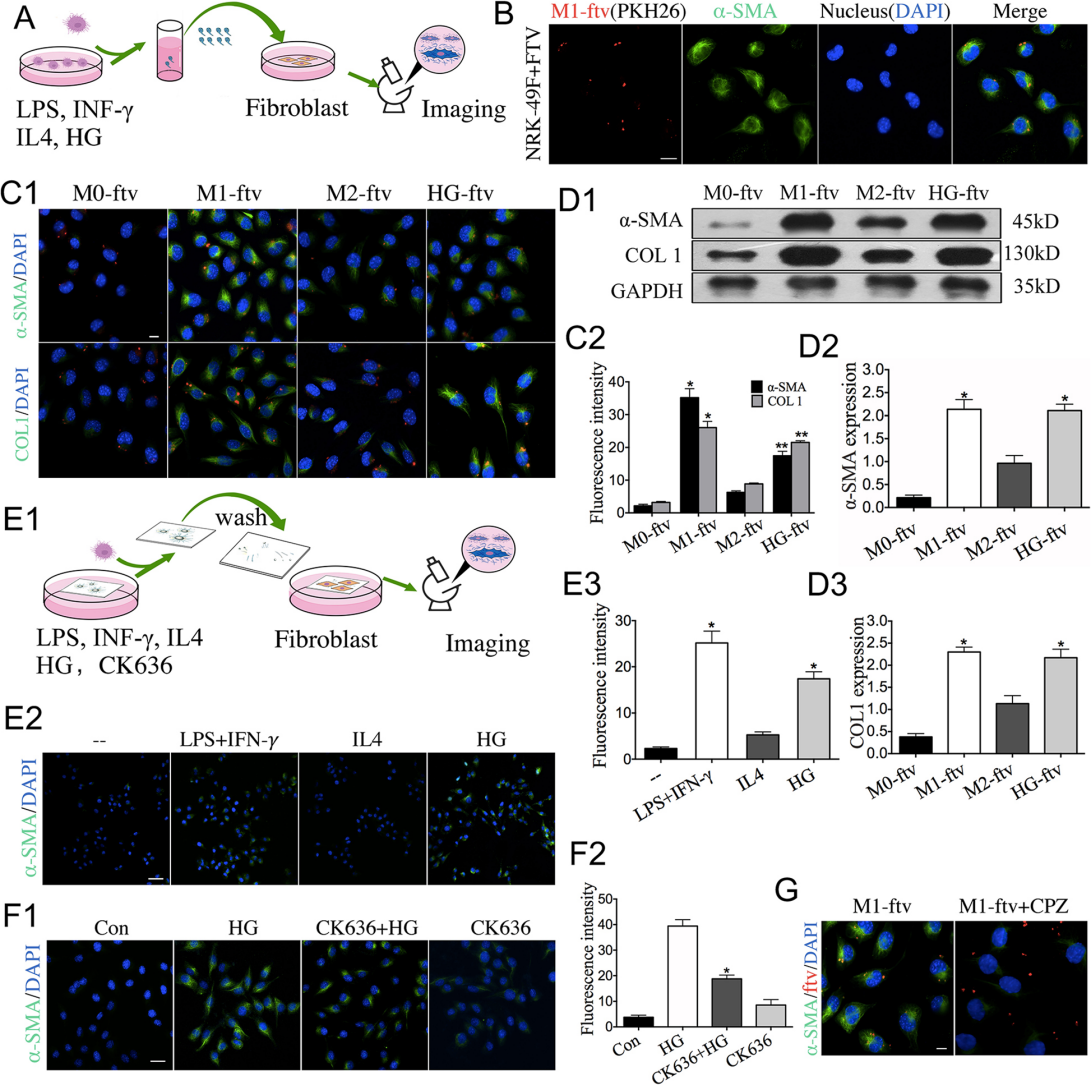
M1-ftv and HG-ftv promote fibroblast transdifferentiation. **A** In vitro experimental plan involving FTV-treated fibroblasts. **B** Confocal images show that PKH26 (red)-labeled M1-ftv are internalized by fibroblasts. *n* = 3. Scale bar:10 μm. **C1** FTV-treated fibroblasts for 24 h followed by α-SMA and COL1 immunostaining. **C2** The mean fluorescent intensities of α-SMA and COL1. *n* = 3. Scale bar: 10 μm. **D1**-**D3** Western blotting to detect the expression of α-SMA and COL1 in FTV-treated fibroblasts group. *n* = 3. **E1 **In vitro experimental plan. **E2** Fibroblasts were incubated on macrophage-experienced coverslips after extensive washing and then subjected to α-SMA immunostaining. **E3** The mean fluorescence intensity. **E4** The relative levels of α-SMA were measured. *n* = 3. Scale bar:10 μm **F1** Macrophage pre-treated CK636, with or without HG, and collected the culture supernatant that contain FTV fractions by slow-speed centrifugation, then fibroblast co-culture with the supernatant and detected by Immunofluorescence. **F2** The mean fluorescent intensities from each group. Scale bar:10 μm. **G** Confocal image of fibroblasts treated with M1-ftv or pre-treated with chlorpromazine (CPZ). *n* = 3. Scale bar: 15 μm. All FTV (red), α-SMA and COL1 (green), and cell nuclei stained with DAPI (blue). *p < 0.05 *vs*. the control group, HG, the M0-ftv

What are the possible clinical implications of these FTV? We hypothesized that FTV might be a potential biological regulator of fibroblast in renal interstitial fibrosis. To evaluate the functional impact of FTV on fibroblast in vitro, RAW264.7 cells were induced into M1 phenotype with LPS in combination with IFN-γ, and M2 phenotype with IL4, or stimulated with HG to produce FTV (named as M1-ftv, M2-ftv, HG-ftv respectively). Then, fibroblasts (NRK-49 F cell lines) were cocultured with PKH26 labelLed FTV for 24 h, after which fibroblast activation markers were examined (Fig. 5A). As shown in Fig. 5B, confocal images showed that FTV were internalized into the fibroblast. Immunofluorescence staining results revealed that the expression levels of α-smooth muscle actin (α-SMA) and Collagen I (COL1), markers of fibroblasts transdifferentiated to myofibroblasts, were significantly increased in the M1-ftv and HG-ftv groups but were expressed at low levels both in M2-ftv and control groups (Fig. 5C1-C2). Consistent with the immunofluorescence analysis results, the result from Western blotting indicated that the expression level of fibroblast transdifferentiation markers in group M1-ftv and HG-ftv were significantly higher than those in the other two groups (Fig. 5D1-D3). Moreover, M1-ftv and HG-ftv specifically induced fibroblasts proliferation after 24 h of coincubation, confirming that fibroblast activation by FTV was accompanied by increase of proliferation (Fig. 5S).

Next, we addressed the functional effects of FTV that had deposited on the macrophage-experienced assay coverslips. RAW264.7 cells were seeded on coverslip, and then stimulated with LPS plus IFN-𝛾, IL4 or HG, After the cell bodies were completely removed by extensive washing of the coverslip surface, FTV-kike particles remained. Fibroblast were incubated on the coverslip (Fig. 5E1). As predicted, fibroblast transdifferentiation occurred on the macrophage-experienced surface. The activation of fibroblast was most significantly enhanced by M1 phenotype and HG treated macrophage-experienced coverslips, but M2 phenotype macrophage-experienced coverslips led to little or minimal fibroblast activation (Fig. 5E2-E3).

To assess whether inhibiting FTV reduces its impact on fibroblast transdifferentiation, we pre-treated macrophages with CK636 to inhibit HG-ftv generation, followed by the collection of culture supernatant containing FTV fractions through low-speed centrifugation. Subsequently, fibroblasts were co-cultured with this supernatant. As shown in Fig. 7F1, the α-SMA expressions of fibroblasts were remarkably decreased in the CK636 pre-treated group. Furthermore, blocking fibroblasts uptake capacity by chlorpromazine (CPZ), a phagocytosis inhibitor, also attenuated these effects (Fig. 5G). These results suggested that HG-ftv reliably involved in fibroblasts activation.

### Macrophage filopodia release IL11-containing FTV

These differential effects on fibroblasts may be partly explained by the different cargo compositions of FTV. FTV gene-profiling was performed to determine which intercellular fibrosis messages might be delivered. Genome-wide expression profiling data have been deposited in the EMBL-EBI ArrayExpress database (accession number: E-MTAB-7994). A total of 2789 (1581 upregulated and 1208 downregulated), 2071 (975 upregulated and 1096 downregulated) and 2450 (1185 upregulated and 1265 downregulated) genes were differentially expressed in M1-ftv, M2-ftv and HG-ftv compared with M0-ftv, respectively. A total of 3869 (2434 u-regulated and 1435 downregulated), 3800 (2296 up-regulated and 1504 downregulated) genes were differentially expressed at M1-ftv and HG-ftv compared with M2-ftv, respectively. These findings support the idea that FTV cargo composition is determined by macrophage status.

**Fig. 6:**
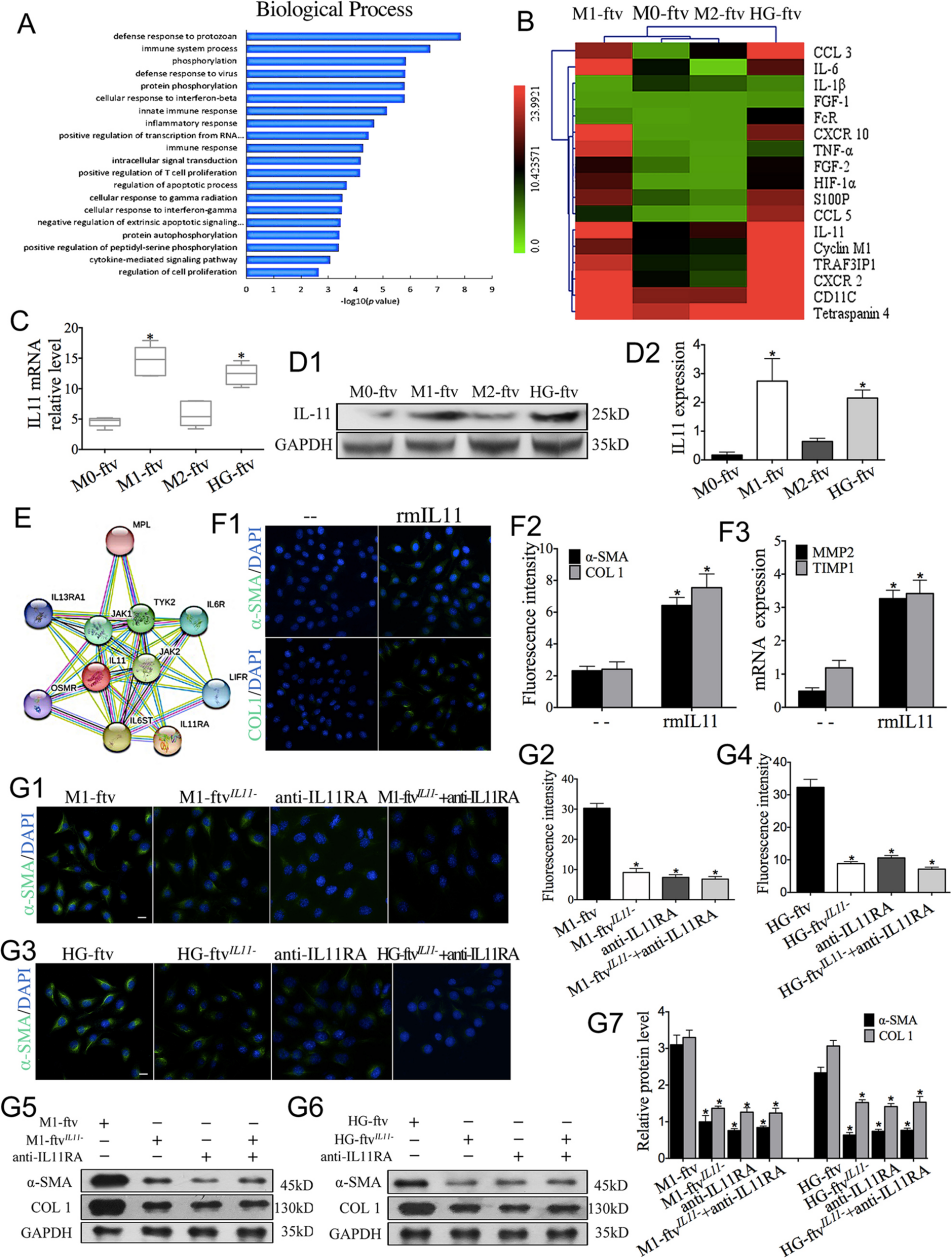
IL11 in HG/M1-ftv drives fibroblast transdifferentiation. **A** M1-ftv Gene ontology and KEGG pathways analysis. **B **The heatmap of FTV Gene profiling.**C **IL11 mRNA quantified using qPCR in each FTV group. *n *= 5. **D1-D2 **IL11 expression detected by Western blotting in each FTV group. *n *= 5. **F** Possible network of IL11. **F1-F2** Immunofluorescence staining of fibroblasts treated with rmIL11 (5 ng/ml) to assess the expression of α-SMA and COL1. **F3 **MMP2 and TIMP1 mRNA quantified using qPCR in each FTV group. *n *= 3. Scale bar: 20 μm. **G1-G4** Immunofluorescence staining of fibroblasts treated with M1-ftv, M1-ftvIL11-, HG-ftv, HG-ftvIL1-, or -combined with anti-IL11 antibody (2 μg/ml) to assess the expression of α-SMA and COL1**.** **G5-G7** α-SMA and COL1 expression detected by Western blotting from each group. *n *= 3. Scale bar: 15 μm. α-SMA and COL1 (green), and cell nuclei stained with DAPI (blue). *P < 0.05 *vs*. the M0-ftv group, the unstimulated (--) group, the M1-ftv the, the HG-ftv group

In the M1-ftv *vs*. M2-ftv comparison, the profile of M1-ftv was unique different from M2-ftv according to Gene Ontology and KEGG pathway analyses, the majority of which were related to the immune response (Fig. 6A). M1-ftv were closely associated with several signalling pathways, including TNF, chemokine, TGF-β and calcium signalling pathways, etc. which could be directly linked to inflammatory response. Hierarchical cluster analysis of differentially expressed transcripts revealed the upregulation of genes. Among the top-ranking genes, we found a specific enrichment of IL11 mRNA in both HG-ftv and M1-ftv groups (Fig. 6B). The elevation of IL11 levels in HG-ftv and M1-ftv were further confirmed by quantitative PCR (Fig. 6C) and western blot analysis (Fig. 6D1-D2). These results suggested that FTV could be used to differentially propagate molecular signals to recipient cells.

IL11 is a close family member of IL6 (Fig. 6E), which was found to be related to he renal fibrosis in our previous studies [30]. Recently, another study has proved that IL11 is also a crucial determinant of cardiovascular fibrosis [31]. Considering that both M1-ftv and HG-ftv actively induce fibroblast transdifferentiation, it is currently unclear whether IL11 present in HG-ftv and M1-ftv can trigger transdifferentiation responses in fibroblasts. We sought to verify the effects of IL11 on fibroblasts. The results showed that the fluorescence of α-SMA and COL1 increased in rmIL11-treated renal fibroblasts, as compared with the results in the control group (Fig. 6F1-F2). Moreover, the expression of MMP2 and TIMP1 was significantly increased in the treatment groups (Fig. 6F3), indicating that IL11 is intricately associated with fibroblast transdifferentiation. 

Moreover, advancing this investigation, fibroblasts were subjected to targeted treatments aimed at disrupting IL11 signalling. This involved the administration of specific anti-IL11RA antibodies individually, as well as a combination treatment involving anti-IL11RA and M1-ftv^*IL11-*^. In comparison to the fibroblast group treated with M1-ftv, the results revealed a striking reduction in the expression levels of α-SMA as shown in (Fig. 6G1-G2). In the HG-ftv^*IL11-*^, HG-ftv^*IL11-*^ combined with anti-IL11RA group, we observed similarly significant reductions of α-SMA expression (Fig. 6G3-G4). Western blot analysis also demonstrated that the silencing of FTV^*IL11*^ leads to a reduction in α-SMA levels, supporting the interaction is dependent on IL11 signals (Fig. 6G5-G7). Overall, these findings suggest the critical role of IL11 signalling in mediating the fibrotic processes initiated by M1/HG-ftv.

### FTV inhibition alleviates renal interstitial fibrosis in diabetic mice

**Fig. 7:**
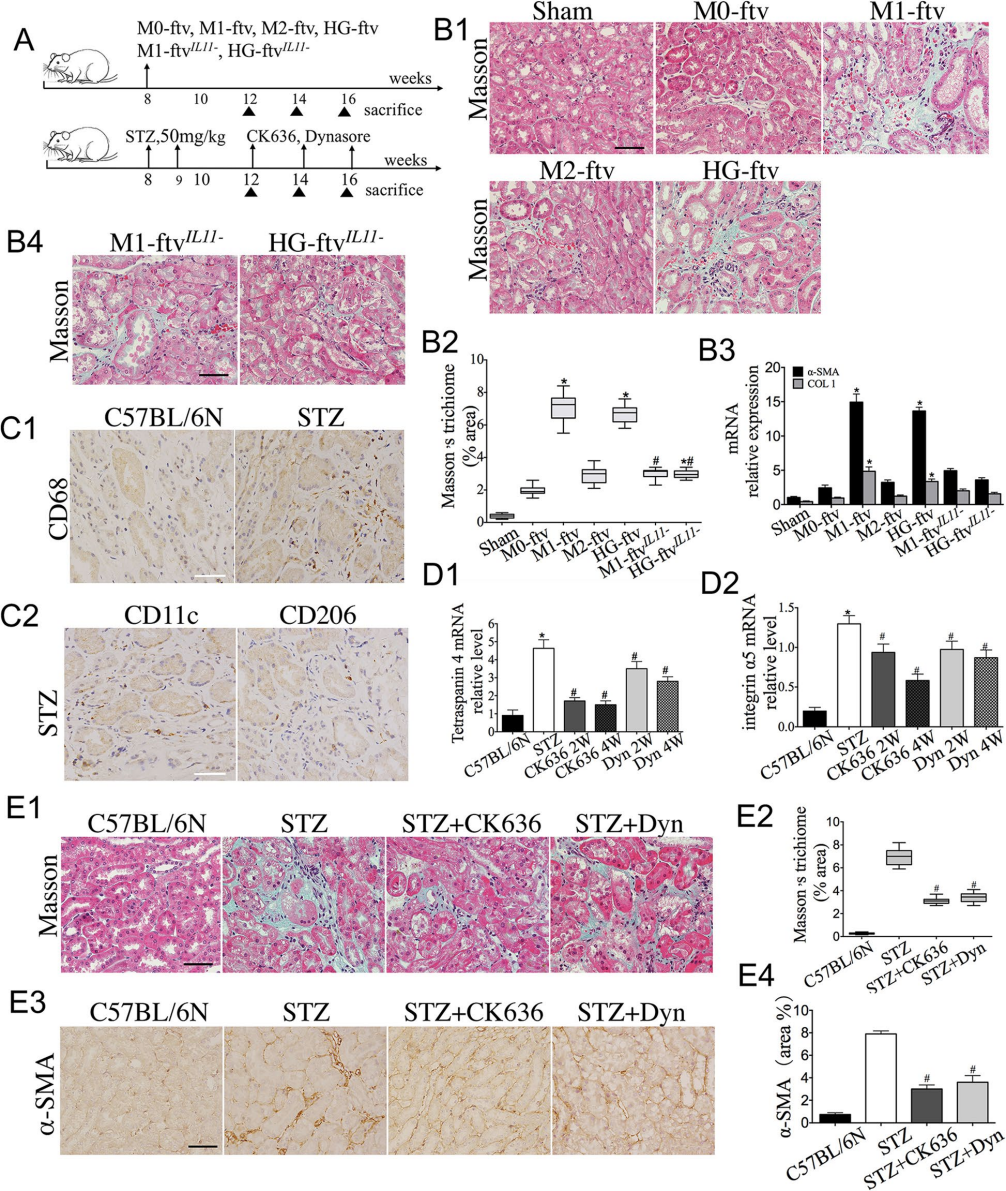
FTV inhibition alleviate renal interstitial fibrosis in diabetic mice. **A** The in vivo treatments plan. **B1**-**B4** M0-ftv, M1-ftv, M2-ftv, HG-ftv, M1-ftv^*IL11-*^ and HG-ftv^*IL11-*^ were transferred into C57/BL6N mice kidney by renal parenchyma injection. Sham mice with PBS injection. Kidney samples were collected, renal tissue sections were stained with Masson’s trichrome, and α-SMA and COL1 in kidney samples was quantified using qPCR in each FTV group. *n* = 5. Scale bar: 50 μm. **C** Immunostaining images of CD68, CD11c, and CD206 in the renal interstitium of C57BL/6 N and STZ mice. *n* = 5. Scale bar: 50 μm. **D1-D2** STZ mice were treated with CK636 or Dynasore. Tetraspanin 4 and integrin α5 in kidney samples detected by RT-qPCR, **E1-E2** and renal tissue sections stained with Masson’s trichrome, **E3-E4** immunostained with α-SMA in each group. *n* = 5. Scale bar: 50 μm.*P < 0.05 *vs*. the Sham group, the C57BL/6 N group. ^*#*^*P* < 0.05 *vs*. the M1-ftv group, the HG-ftv group, the STZ group

To investigate the potential role of FTV in renal interstitial fibrosis, we administered isolated FTV to mice to determine whether FTV could induce renal interstitial fibrosis. The in vivo experimental plan is illustrated in Fig. 7A. Recipient mice were injected with M0-ftv, M1-ftv, M2-ftv and HG-ftv via direct injection (200 µl) to the mice kidney. Sham mice were injected with the same volume of PBS. After 2 weeks of injection, kidneys from mice in the M1-ftv and HG-ftv groups showed an evident increase of collagen content deposition according to Masson trichrome staining, while mice in the M2-ftv group exhibited minimal interstitial fibrosis records. M0-ftv was largely ineffective in mouse kidney fibrosis and no fibrosis change was found in sham mice (Fig. 7B-B2). Similarly, in the HG-ftv and M1-ftv treatment group, the expression of α-SMA and COL1 significantly increased compared to that in the M2-ftv group (Fig. 7B3).

Given IL11enriched in HG-ftv and M1-ftv functions as a profibrotic factor, we determined whether the transfer of IL11 by HG-ftv and M1-ftv contributes to the development of renal interstitial fibrosis. IL11 specific siRNAs was used to generate the HG-ftv^*IL11-*^ and M1-ftv ^*IL11-*^, which were directly injected to the mice kidney. After 2 weeks of injection, histologic examination revealed that the IL11 deficient FTV induced less severe renal interstitial fibrosis (Fig. 7B4). Similar results were obtained when fibrosis markers levels were measured by RT-PCR analysis (Fig. 7B3). These results imply a strong correlation between HG/M1-ftv and diabetic renal fibrosis.

DN is an inflammatory disease, and recent studies have identified a primary relationship between macrophage accumulation and renal fibrosis in DN [32, 33]. Together with our findings of FTV both in vivo and vitro, these findings prompted us to investigate the pathophysiological relevance of FTV in renal interstitial fibrosis of DN. We revealed that renal interstitium of diabetic C57BL/6 N mice showed an increase of inflammatory macrophage (CD68^+^ cells) in comparison to control mice (Fig. 7C1). The number of M2 macrophages (CD206^+^ cells) in the inflammatory site was less than M1 macrophages (CD11C^+^ cells) (Fig. 7C2), implying tha M1 macrophage activation correlate with the severity of early tubulointerstitial damage. Furthermore, we observed that the expression of FTV markers Tetraspanin 4 and integrin α5 was higher in kidney from DN mice compared with the control (Fig. 7D1-D2). However, it cannot be entirely ruled out that other cells may also express Tetraspanin 4 and integrin α5, these findings suggest a potential association between FTV and renal interstitial fibrosis in DN.

To address whether FTV are involved in renal interstitial fibrosis in DN, an FTV suppression strategy was applied in model mice. The in vivo treatments plan as shown in Fig. 7A. STZ mice were treated with FTV inhibitor CK636 or Dynasore, leading to reduced collagen deposition, as observed in Masson's trichrome staining (Fig. 7E1-E2). Similarly, assessment of α-SMA assessment confirmed the decrease of renal interstitial fibrosis compared with that in the STZ group (Fig. 7E3-E4). Moreover, we noticed that the expression levels of Tetraspanin 4 and integrin α5 were decreased in the kidney as early as 2 weeks after CK636 or Dynasore treatment (Fig. 7D1-D2). These experiments indicated that the FTV inhibitor represses renal interstitial fibrosis of DN probably by targeting FTV.

**Fig. 8:**
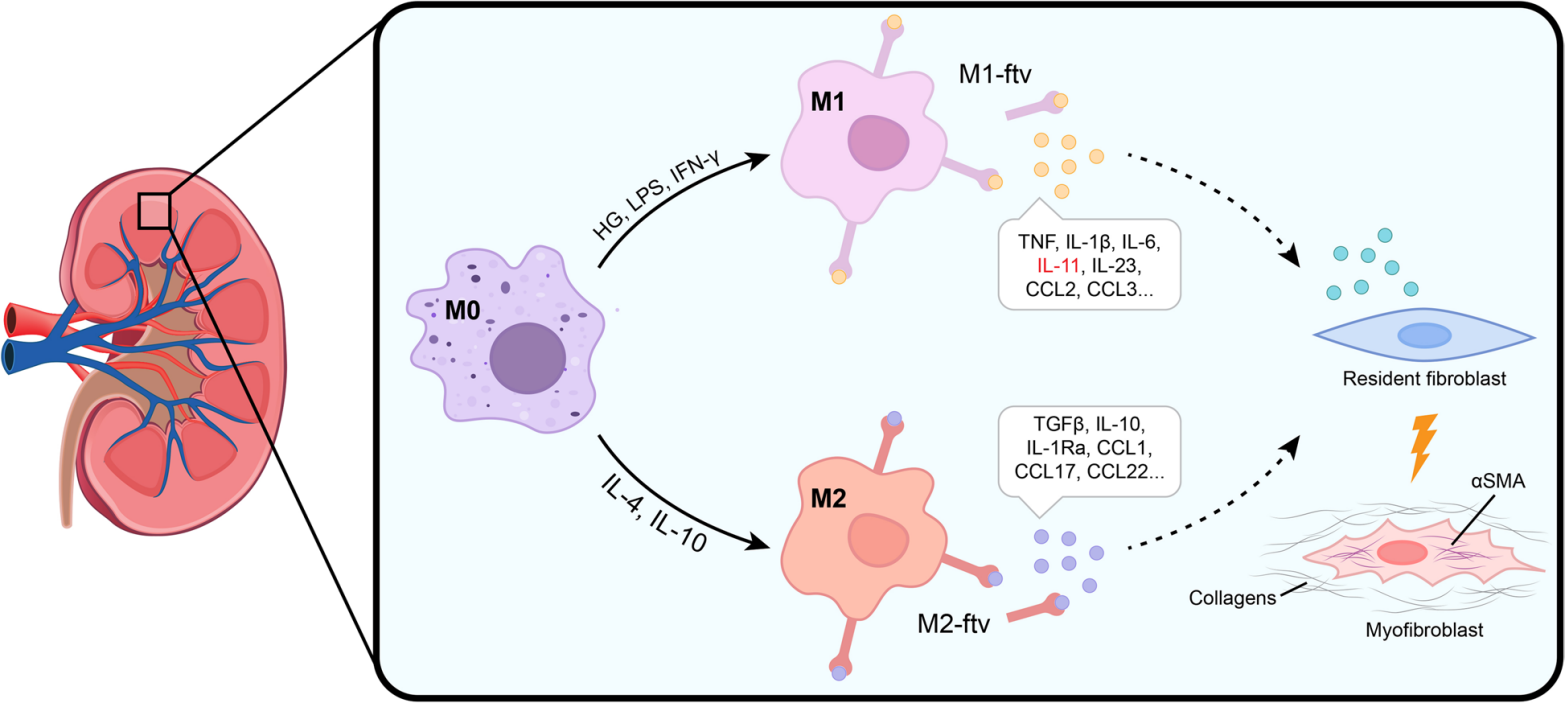
Schematic diagram illustrating fibroblast transdifferentiation induced by the HG/M1-ftvIL11 signal originating from macrophage filopodia

## Discussion

Macrophages are key factors in the initiation and propagation of kidney disease [16]. Traditionally, activated macrophages secrete a broad range of inflammatory cytokines, chemokines and other soluble mediators, which are believed to result in target organ dysfunction and fibrosis, such as pulmonary fibrosis and renal fibrosis in DN [34, 35]. Macrophage filopodia are long, thin, finger-like protrusions, and much is known about its roles in pathogen clearance, however, we have recently documented filopodia tip vesicle-like structure involving cell–cell communication in a highly specific manner. The term " filopodial tip vesicles, FTV” is used to describe the vesicle-like structure based on its subcellular features. With the purpose to highlight the origin of FTV from macrophage filopodia and the special role of filopodia-mediated intercellular communication. FTV fall into EV category, but are distinguished from classical EVs. FTV could be a represent highly conserved manner exploited by macrophage as a mode of intercellular communication. This observation is of some interest because FTV convey functional regulatory molecule signals to renal fibroblast. IL11 enriched in HG/M1-ftv driving fibroblasts transdifferentiation (Fig. 8), and this may be a novel mechanism underlying renal interstitial fibrosis in DN.

Life depends on the ability of cells to communicate with each other. Much of this crosstalk occurs at cell–cell contacts and is regulated by complex structural interfaces [36]. Macrophages contain bundles of filopodia, which are very dynamic cellular structures, that can grow and retract, push or pull in response to chemoattractive signals [1]. In the current study, we first visualized the formation and release of some particular vesicle-like structures from the tip of macrophage filopodia. To date, this structure has not been reported in literature. Most of cells release extracellular vesicle, such as exosomes (50 ~ 100 nm in diameter), microvesicles (100 ~ 1000 nm in diameter) and apoptotic bodies, which represent universal mechanism by which cells perform intercellular communication [37]. For example, exosome formed by the inward budding of endosomal membrane and secreted upon fusion of multivesicular endosomes with the cell surface [38]. Microvesicles are generated by the outward budding and fission of the plasma membrane [39], and apoptotic bodies shed from dying cells [40]. Some other vesicles such as oncosomes, spheresomes are comparable with FTV in size [28, 41], however, they are produced by budding and different from FTV in genesis. FTV is a filopodia-dependent membrane vesicle that initially connects to the filopodial membrane. It seems that macrophage filopodia undergo retraction or sweeping leading to the dissociation.

More recently, researchers have identified some cellar fibers derived vesicles including migrasome, exophers, and neutrophil trails, that primarily implicated in intercellular communication [2, 19, 42]. Migrasome have been described as exosome‑like vesicles, but unlike exosomes, these arise from the disintegration of the retraction fibers of a migrating cell [19, 43]. As in the present report, FTV in some like manner derive from nanotubular filopodia and also express Tetraspanin 4 and integrin α5, which are markers of migrasome. In this respect, it is highly likely that FTV falls within the category of migrasome. As we describe ultrafine structures by EM, FTV are derived from the tips filopodia. Unlike the FTV, neutrophil trails are neutrophil long membrane tethers that extend from their elongated uropods during migration. Exophers are generated from neurons in *C. elegans*, which is different from the generation of FTV, however, exophers are much larger in volume (1000–7000 nm in diameter) and harbour protein aggregates and organelles, including lysosomes and mitochondria, and we did not observe such big organelles in FTV. In cell biology, these new fiber derived vesicles function in cell–cell signalling and intercellular transport that actively contribute to neuronal network formation, innate or adaptive immunity, and chronic inflammatory diseases [44, 45]. Their close relationship implies that FTV may have primary functions.

There has been substantial interest in the mechanisms by which filopodia mediate distal interactions between cells. Via the filopodia, integral membrane proteins such as MHC-I are transferred between macrophages and HeLa cells [46]. This report provides new insights into the role of macrophage filopodia in cell–cell communication. At the ultrastructural level, the uptake of FTV by neighbour renal fibroblasts were observed both in renal interstitium of DN mice and patients. Furthermore, FTV could be highly efficient and capable of signal transfer due to their unique structure and large in size. Considering FTV functions and possible applications, FTV can initiate fibroblast transdifferentiation and induce renal interstitial fibrosis in mice, and FTV inhibitors can attenuate renal interstitial fibrosis in STZ model mice. Thus, the FTV-driven fibrogenic response may serve as a potential mechanism underlying renal interstitial fibrosis in DN.

Macrophages are highly heterogeneous cells and exhibit distinct functions in response to stimuli in the microenvironment [47]. In response to LPS/IFN-γ or HG, macrophages undergo M1 polarization, which can trigger renal fibrosis by activating fibroblasts [48]. Recently, LPS was reported to increase the dynamics and formation of filopodia [29], raising questions about whether stimulation modulates FTV formation. We observed that LPS/IFN-γ, IL4 or HG enhanced FTV production. When filopodia were blocked by the dynamin inhibitor Dynasore or actin filament inhibitor CK-636, FTV production greatly decreased, which is similar to migrasome. Of note, M1-ftv and HG-ftv, but not M2-ftv, induced fibroblasts transdifferentiation, suggesting that FTV from macrophages of different phenotypes may exhibit distinct functional properties. This also highlights that HG-ftv and M1-ftv may share some cargo in terms of molecular composition or signalling pathway components.

As an important IL6 family member, IL11 was discovered due to its association with haematopoiesis and tumorigenesis, but it was recently found to be crucial for cardiovascular fibrosis [31, 49, 50]. In the current study, IL11 was proven to efficiently affect on renal fibroblasts. Interestingly, HG-ftv and M1-ftv are rich in IL11.Knockout of IL11 in M1-ftv or blockade of fibroblast IL11R decreased fibroblast transdifferentiation in vitro, and FTV^*IL11−*^ induced less severe renal interstitial fibrosis in vivo. As large vesicular structures, FTV are probably similar classical EVs and cytoneme structures that carry selective mediators to recipient cells [51, 52]. As a small and diffusible molecule, IL11 could diffuse into the renal interstitium and its targeting effects may not be sufficiently efficient. However, FTV serve as large carriers of IL11, and quantities of IL11 could be preserved and transferred to remote fibroblasts in a highly efficient manner.

Our understanding of FTV in this study is still limited. Further research will be conducted to characterize the features, markers, cargo composition, and biogenesis of FTV. In addition, FTV cargo included components such as proteins, nucleic acids, lipids, metabolic products, it cannot be excluded that other unconfirmed molecules in FTV may be responsible for the profibrotic effects. In future research, more depth of structural and functional roles of FTV shall be delineated.

In summary, this work highlights the complex functional mechanisms of macrophage filopodia. For macrophages, FTV-derived message transfer might be a specific mechanism of communication to target cells. FTV induce fibroblast transdifferentiation by transferring IL11, which provides new insights into the mechanisms underlying renal interstitial fibrosis in DN.

### Supplementary Information

**Additional file 1.**

**Additional file 2.**

**Additional file 3.**

**Additional file 4.**

**Additional file 5.**

**Additional file 6.**

## Data Availability

All the data needed to evaluate the conclusions in the manuscript are presented in the manuscript.
