# Supplementary material for: Macrophages release IL11-containing filopodial tip vesicles and contribute to renal interstitial inflammation
Source: Cell Commun Signal. 2023 Oct 18;21:293. doi: 10.1186/s12964-023-01327-6 (PMC10585809; doi:10.1186/s12964-023-01327-6)
Supplement: Supplementary file 4 — Additional file 3. [file 12964_2023_1327_MOESM3_ESM.pdf]

载体概况

载体ID	VB180422-1013vqa
载体名称	pLV[Exp]-EGFP:T2A:Puro-EF1A>mTspan4[NM_001252588.1]
创建日期	2018-04-22
载体大小	10091 bp
载体类型	Lentivirus gene expression vector (3rd generation)
启动子	EF1A
ORF	mTspan4[NM_001252588.1]
Marker	EGFP:T2A:Puro
质粒拷贝数	High
抗生素抗性	Ampicillin
克隆宿主菌系	Stbl3 (or alternative strain)

载体图谱

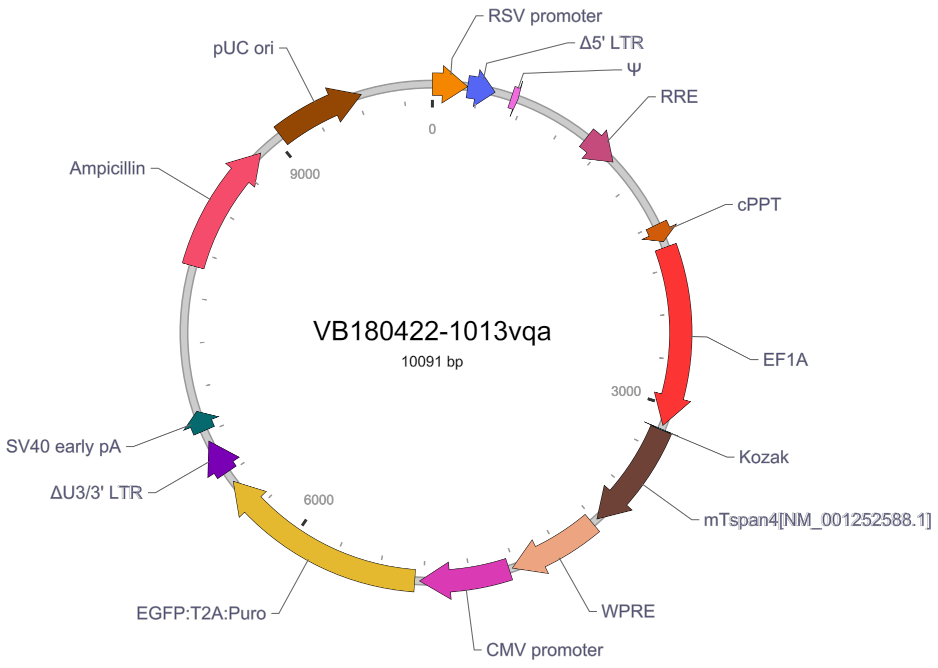

载体元件

名称	位点	大小 (bp)	类型	描述	应用注释
RSV promoter	1-229	229	Promoter	Rous sarcoma virus enhancer/promoter	Strong promoter; drives transcription of viral RNA in packaging cells.
Δ5' LTR	230-410	181	LTR	Truncated HIV-1 5' long terminal repeat	Allows transcription of viral RNA and its packaging into virus.
Ψ	521-565	45	Miscellaneous	HIV-1 packaging signal	Allows packaging of viral RNA into virus.
RRE	1075-1308	234	Miscellaneous	HIV-1 Rev response element	Rev protein binding site that allows Rev-dependent nuclear export of viral RNA during viral packaging.

名称	位点	大小 (bp)	类型	描述	应用注释
cPPT	1803-1920	118	Miscellaneous	Central polypurine tract	Facilitates the nuclear import of HIV-1 cDNA through a central DNA flap.
**EF1A**	1959-3137	1179	Promoter	Human eukaryotic translation elongation factor 1 α1 promoter	Strong promoter.
Kozak	3162-3167	6	Miscellaneous	Kozak translation initiation sequence	Facilitates translation initiation of ATG start codon downstream of the Kozak sequence.
**mTspan4[NM_001252588.1]**	3168-3884	717	ORF	*None*	*None*
WPRE	3923-4520	598	Miscellaneous	Woodchuck hepatitis virus posttranscriptional regulatory element	Enhances viral RNA stability in packaging cells, leading to higher titer of packaged virus.
CMV promoter	4542-5129	588	Promoter	Human cytomegalovirus immediate early enhancer/promoter	Strong promoter; may have variable strength in some cell types.
**EGFP:T2A:Puro**	5161-6540	1380	ORF	EGFP and Puro linked by T2A	Allows cells to be visualized by green fluorescence and resistant to puromycin.
ΔU3/3' LTR	6611-6845	235	LTR	Truncated HIV-1 3' long terminal repeat	Allows packaging of viral RNA into virus; self-inactivates the 5' LTR by a copying mechanism during viral genome integration; contains polyadenylation signal for transcription termination.
SV40 early pA	6918-7052	135	PolyA_signal	Simian virus 40 early polyadenylation signal	Allows transcription termination and polyadenylation of mRNA transcribed by Pol II RNA polymerase.
Ampicillin	8006-8866	861	ORF	Ampicillin resistance gene	Allows E. coli to be resistant to ampicillin.
pUC ori	9037-9625	589	Rep_origin	pUC origin of replication	Facilitates plasmid replication in E. coli; regulates high-copy plasmid number (500-700).

载体序列

```
   1 AATGTAGTCT TATGCAATAC TCTTGTAGTC TTGCAACATG GTAACGATGA GTTAGCAACA TGCCTTACAA GGAGAGAAAA AGCACCGTGC ATGCCGATTG
 101 GTGGAAGTAA GGTGGTACGA TCGTGCCTTA TTAGGAAGGC AACAGACGGG TCTGACATGG ATTGGACGAA CCACTGAATT GCCGCATTGC AGAGATATTG
 201 TATTTAAGTG CCTAGCTCGA TACATAAACG GGTCTCTCTG GTTAGACCAG ATCTGAGCCT GGGAGCTCTC TGGCTAACTA GGGAACCCAC TGCTTAAGCC
 301 TCAATAAAGC TTGCCTTGAG TGCTTCAAGT AGTGTGTGCC CGTCTGTTGT GTGACTCTGG TAACTAGAGA TCCCTCAGAC CCTTTTAGTC AGTGTGGAAA
 401 ATCTCTAGCA GTGGCGCCCG AACAGGGACT TGAAAGCGAA AGGGAAACCA GAGGAGCTCT CTCGACGCAG GACTCGGCTT GCTGAAGCGC GCACGGCAAG
 501 AGGCGAGGGG CGGCGACTGG TGAGTACGCC AAAAATTTTG ACTAGCGGAG GCTAGAAGGA GAGAGATGGG TGCGAGAGCG TCAGTATTAA GCGGGGGAGA
 601 ATTAGATCGC GATGGGAAAA AATTCGGTTA AGGCCAGGGG GAAAGAAAAA ATATAAATTA AAACATATAG TATGGGCAAG CAGGGAGCTA GAACGATTCG
 701 CAGTTAATCC TGGCCTGTTA GAAACATCAG AAGGCTGTAG ACAAATACTG GGACAGCTAC AACCATCCCT TCAGACAGGA TCAGAAGAAC TTAGATCATT
 801 ATATAATACA GTAGCAACCC TCTATTGTGT GCATCAAAGG ATAGAGATAA AAGACACCAA GGAAGCTTTA GACAAGATAG AGGAAGAGCA AAACAAAAGT
 901 AAGACCACCG CACAGCAAGC GGCCGCTGAT CTTCAGACCT GGAGGAGGAG ATATGAGGGA CAATTGGAGA AGTGAATTAT ATAAATATAA AGTAGTAAAA
1001 ATTGAACCAT TAGGAGTAGC ACCCACCAAG GCAAAGAGAA GAGTGGTGCA GAGAGAAAAA AGAGCAGTGG GAATAGGAGC TTTGTTCCTT GGGTTCTTGG
1101 GAGCAGCAGG AAGCACTATG GGCGCAGCGT CAATGACGCT GACGGTACAG GCCAGACAAT TATTGTCTGG TATAGTGCAG CAGCAGAACA ATTTGCTGAG
1201 GGCTATTGAG GCGCAACAGC ATCTGTTGCA ACTCACAGTC TGGGGCATCA AGCAGCTCCA GGCAAGAATC CTGGCTGTGG AAAGATACCT AAAGGATCAA
1301 CAGCTCCTGG GGATTTGGGG TTGCTCTGGA AAACTCATTT GCACCACTGC TGTGCCTTGG AATGCTAGTT GGAGTAATAA ATCTCTGGAA CAGATTTGGA
1401 ATCACACGAC CTGGATGGAG TGGGACAGAG AAATTAACAA TTACACAAGC TTAATACACT CCTTAATTGA AGAATCGCAA AACCAGCAAG AAAAGAATGA
1501 ACAAGAATTA TTGGAATTAG ATAAATGGGC AAGTTTGTGG AATTGGTTTA ACATAACAAA TTGGCTGTGG TATATAAAAT TATTCATAAT GATAGTAGGA
1601 GGCTTGGTAG GTTTAAGAAT AGTTTTTGCT GTACTTTCTA TAGTGAATAG AGTTAGGCAG GGATATTCAC CATTATCGTT TCAGACCCAC CTCCCAACCC
1701 CGAGGGGACC CGACAGGCCC GAAGGAATAG AAGAAGAAGG TGGAGAGAGA GACAGAGACA GATCCATTCG ATTAGTGAAC GGATCTCGAC GGTATCGCTA
1801 GCTTTTAAAA GAAAAGGGGG GATTGGGGGG TACAGTGCAG GGGAAAGAAT AGTAGACATA ATAGCAACAG ACATACAAAC TAAAGAATTA CAAAAACAAA
1901 TTACAAAAAT TCAAAATTTT ACTAGTGATT ATCGGATCAA CTTTGTATAG AAAAGTTGGG CTCCGGTGCC CGTCAGTGGG CAGAGCGCAC ATCGCCCACA
2001 GTCCCCGAGA AGTTGGGGGG AGGGGTCGGC AATTGAACCG GTGCCTAGAG AAGGTGGCGC GGGGTAAACT GGGAAAGTGA TGTCGTGTAC TGGCTCCGCC
2101 TTTTTCCCGA GGGTGGGGGA GAACCGTATA TAAGTGCAGT AGTCGCCGTG AACGTTCTTT TTCGCAACGG GTTTGCCGCC AGAACACAGG TAAGTGCCGT
2201 GTGTGGTTCC CGCGGGCCTG GCCTCTTTAC GGGTTATGGC CCTTGCGTGC CTTGAATTAC TTCCACCTGG CTGCAGTACG TGATTCTTGA TCCCGAGCTT
```

2301 CGGGTTGGAA GTGGGTGGGA GAGTTCGAGG CCTTGCGCTT AAGGAGCCCC TTCGCCTCGT GCTTGAGTTG AGGCCTGGCC TGGGCGCTGG GGCCGCCGCG
2401 TGCGAATCTG GTGGCACCTT CGCGCCTGTC TCGCTGCTTT CGATAAGTCT CTAGCCATTT AAAATTTTTG ATGACCTGCT GCGACGCTTT TTTTCTGGCA
2501 AGATAGTCTT GTAAATGCGG GCCAAGATCT GCACACTGGT ATTTCGGTTT TTGGGGCCGC GGGCGGCGAC GGGGCCCGTG CGTCCCAGCG CACATGTTCG
2601 GCGAGGCGGG GCCTGCGAGC GCGGCCACCG AGAATCGGAC GGGGGTAGTC TCAAGCTGGC CGGCCTGCTC TGGTGCCTGG TCTCGCGCCG CCGTGTATCG
2701 CCCCGCCCTG GGCGGCAAGG CTGGCCCGGT CGGCACCAGT TGCGTGAGCG GAAAGATGGC CGCTTCCCGG CCCTGCTGCA GGGAGCTCAA AATGGAGGAC
2801 GCGGCGCTCG GGAGAGCGGG CGGGTGAGTC ACCCACACAA AGGAAAAGGG CCTTTCCGTC CTCAGCCGTC GCTTCATGTG ACTCCACGGA GTACCGGGCG
2901 CCGTCCAGGC ACCTCGATTA GTTCTCGAGC TTTTGGAGTA CGTCGTCTTT AGGTTGGGGG GAGGGGTTTT ATGCGATGGA GTTTCCCCAC ACTGAGTGGG
3001 TGGAGACTGA AGTTAGGCCA GCTTGGCACT TGATGTAATT CTCCTTGGAA TTTGCCCTTT TTGAGTTTGG ATCTTGGTTC ATTCTCAAGC CTCAGACAGT
3101 GGTTCAAAGT TTTTTTCTTC CATTTCAGGT GTCGTGACAA GTTTGTACAA AAAAGCAGGC TGCCACCATG GCGCGCGGCT GCCTCCAGGG CGTCAAGTAC
3201 CTCATGTTCG CCTTCAACCT GCTCTTCTGG CTGGGTGGCT GTGGTGTCCT GGGTGTTGGC ATCTGGTTGG CTGCCACACA GGGAAACTTT GCCACCTTAT
3301 CATCCTCATT TCCATCCTTG TCGGCTGCCA ACCTGCTCAT CGTCACCGGG ACCTTCGTCA TGGCCATCGG CTTCGTGGGC TGCATTGGGG CCCTCAAGGA
3401 GAACAAGTGC CTACTGCTCA CTTTCTTTGT GCTGCTGCTG CTAGTGTTCC TGCTGGAAGC CACCATTGCT GTGCTCTTCT TTGCCTACAG TGACAAGATT
3501 GACAGTTATG CCCAACAAGA CCTGAAGAAG GGCCTGCATC TGTATGGCAC ACAGGGCAAC GTGGGCCTCA CCAATGCCTG GAGCATCATC CAGACTGATT
3601 TCCGATGCTG TGGAGTTTCC AATTACACTG ATTGGTTTGA GGTATACAAT GCCACTCGTG TGCCTGACTC CTGCTGTCTG GAGTTCAGTG ATAGCTGTGG
3701 GTTACATGAA CCTGGTACCT GGTGGAAGTC GCCCTGTTAT GAGACAGTGA AGGCCTGGCT CCAGGAGAAC CTGCTAGCTG TGGGCATCTT TGGACTGTGC
3801 ACGGCACTGG TGCAGATTCT GGGCCTCACC TTCGCTATGA CCATGTACTG CCAGGTGGTA AAGGCGGACA CCTACTGTGC ATAGACCCAG CTTTCTTGTA
3901 CAAAGTGGTG ATAATCGAAT TCCGATAATC AACCTCTGGA TTACAAAATT TGTGAAAGAT TGACTGGTAT TCTTAACTAT GTTGCTCCTT TTACGCTATG
4001 TGGATACGCT GCTTTAATGC CTTTGTATCA TGCTATTGCT TCCCGTATGG CTTTCATTTT CTCCTCCTTG TATAAATCCT GGTTGCTGTC TCTTTATGAG
4101 GAGTTGTGGC CCGTTGTCAG GCAACGTGGC GTGGTGTGCA CTGTGTTTGC TGACGCAACC CCCACTGGTT GGGGCATTGC CACCACCTGT CAGCTCCTTT
4201 CCGGGACTTT CGCTTTCCCC CTCCCTATTG CCACGGCGGA ACTCATCGCC GCCTGCCTTG CCCGCTGCTG GACAGGGGCT CGGCTGTTGG GCACTGACAA
4301 TTCCGTGGTG TTGTCGGGGA AGCTGACGTC CTTTCCATGG CTGCTCGCCT GTGTTGCCAC CTGGATTCTG CGCGGGACGT CCTTCTGCTA CGTCCCTTCG
4401 GCCCTCAATC CAGCGGACCT TCCTTCCCGC GGCCTGCTGC CGGCTCTGCG GCCTCTTCCG CGTCTTCGCC TTCGCCCTCA GACGAGTCGG ATCTCCCTTT
4501 GGGCCGCCTC CCCGCATCGG GAATTCCCGC GGTTCGAACG CGTTGACATT GATTATTGAC TAGTTATTAA TAGTAATCAA TTACGGGGTC ATTAGTTCAT
4601 AGCCCATATA TGGAGTTCCG CGTTACATAA CTTACGGTAA ATGGCCCGCC TGGCTGACCG CCCAACGACC CCCGCCCATT GACGTCAATA ATGACGTATG
4701 TTCCCATAGT AACGCCAATA GGGACTTTCC ATTGACGTCA ATGGGTGGAG TATTTACGGT AAACTGCCCA CTTGGCAGTA CATCAAGTGT ATCATATGCC
4801 AAGTACGCCC CCTATTGACG TCAATGACGG TAAATGGCCC GCCTGGCATT ATGCCCAGTA CATGACCTTA TGGGACTTTC CTACTTGGCA GTACATCTAC
4901 GTATTAGTCA TCGCTATTAC CATGGTGATG CGGTTTTGGC AGTACATCAA TGGGCGTGGA TAGCGGTTTG ACTCACGGGG ATTTCCAAGT CTCCACCCCA
5001 TTGACGTCAA TGGGAGTTTG TTTTGGCACC AAAATCAACG GGACTTTCCA AAATGTCGTA ACAACTCCGC CCCATTGACG CAAATGGGCG GTAGGCGTGT
5101 ACGGTGGGAG GTCTATATAA GCAGAGCTCT CTGGCTAACT AGAGAACCCA CTGCGCCACC ATGGTGAGCA AGGGCGAGGA GCTGTTCACC GGGGTGGTGC
5201 CCATCCTGGT CGAGCTGGAC GGCGACGTAA ACGGCCACAA GTTCAGCGTG TCCGGCGAGG GCGAGGGCGA TGCCACCTAC GGCAAGCTGA CCCTGAAGTT
5301 CATCTGCACC ACCGGCAAGC TGCCCGTGCC CTGGCCCACC CTCGTGACCA CCCTGACCTA CGGCGTGCAG TGCTTCAGCC GCTACCCCGA CCACATGAAG
5401 CAGCACGACT TCTTCAAGTC CGCCATGCCC GAAGGCTACG TCCAGGAGCG CACCATCTTC TTCAAGGACG ACGGCAACTA CAAGACCCGC GCCGAGGTGA
5501 AGTTCGAGGG CGACACCCTG GTGAACCGCA TCGAGCTGAA GGGCATCGAC TTCAAGGAGG ACGGCAACAT CCTGGGGCAC AAGCTGGAGT ACAACTACAA
5601 CAGCCACAAC GTCTATATCA TGGCCGACAA GCAGAAGAAC GGCATCAAGG TGAACTTCAA GATCCGCCAC AACATCGAGG ACGGCAGCGT GCAGCTCGCC
5701 GACCACTACC AGCAGAACAC CCCCATCGGC GACGGCCCCG TGCTGCTGCC CGACAACCAC TACCTGAGCA CCCAGTCCGC CCTGAGCAAA GACCCCAACG
5801 AGAAGCGCGA TCACATGGTC CTGCTGGAGT TCGTGACCGC CGCCGGGATC ACTCTCGGCA TGGACGAGCT GTACAAGGGC TCCGGAGAGG GCAGGGGAAG
5901 TCTTCTAACA TGCGGGGACG TGGAGGAAAA TCCCGGCCCC ATGACCGAGT ACAAGCCCAC GGTGCGCCTC GCCACCCGCG ACGACGTCCC CAGGGCCGTA
6001 CGCACCCTCG CCGCCGCGTT CGCCGACTAC CCCGCCACGC GCCACACCGT CGATCCGGAC CGCCACATCG AGCGGGTCAC CGAGCTGCAA GAACTCTTCC
6101 TCACGCGCGT CGGGCTCGAC ATCGGCAAGG TGTGGGTCGC GGACGACGGC GCCGCGGTGG CGGTCTGGAC CACGCCGGAG AGCGTCGAAG CGGGGGCGGT
6201 GTTCGCCGAG ATCGGCCCGC GCATGGCCGA GTTGAGCGGT TCCCGGCTGG CCGCGCAGCA ACAGATGGAA GGCCTCCTGG CGCCGCACCG GCCCAAGGAG
6301 CCCGCGTGGT TCCTGGCCAC CGTCGGCGTC TCGCCCGACC ACCAGGGCAA GGGTCTGGGC AGCGCCGTCG TGCTCCCCGG AGTGGAGGCG GCCGAGCGCG
6401 CCGGGGTGCC CGCCTTCCTG GAGACCTCCG CGCCCCGCAA CCTCCCCTTC TACGAGCGGC TCGGCTTCAC CGTCACCGCC GACGTCGAGG TGCCCGAAGG
6501 ACCGCGCACC TGGTGCATGA CCCGCAAGCC CGGTGCCTGA GGTACCTTTA AGACCAATGA CTTACAAGGC AGCTGTAGAT CTTAGCCACT TTTTAAAAGA
6601 AAAGGGGGGA CTGGAAGGGC TAATTCACTC CCAACGAAGA CAAGATCTGC TTTTTGCTTG TACTGGGTCT CTCTGGTTAG ACCAGATCTG AGCCTGGGAG
6701 CTCTCTGGCT AACTAGGGAA CCCACTGCTT AAGCCTCAAT AAAGCTTGCC TTGAGTGCTT CAAGTAGTGT GTGCCCGTCT GTTGTGTGAC TCTGGTAACT
6801 AGAGATCCCT CAGACCCTTT TAGTCAGTGT GGAAAATCTC TAGCAGTAGT AGTTCATGTC ATCTTATTAT TCAGTATTTA TAACTTGCAA AGAAATGAAT
6901 ATCAGAGAGT GAGAGGAACT TGTTTATTGC AGCTTATAAT GGTTACAAAT AAAGCAATAG CATCACAAAT TTCACAAATA AAGCATTTTT TTCACTGCAT
7001 TCTAGTTGTG GTTTGTCCAA ACTCATCAAT GTATCTTATC ATGTCTGGCT CTAGCTATCC CGCCCCTAAC TCCGCCCATC CCGCCCCTAA CTCCGCCCAG
7101 TTCCGCCCAT TCTCCGCCCC ATGGCTGACT AATTTTTTTT ATTTATGCAG AGGCCGAGGC CGCCTCGGCC TCTGAGCTAT TCCAGAAGTA GTGAGGAGGC
7201 TTTTTTGGAG GCCTAGGGAC GTACCCAATT CGCCCTATAG TGAGTCGTAT TACGCGCGCT CACTGGCCGT CGTTTTACAA CGTCGTGACT GGGAAAACCC
7301 TGGCGTTACC CAACTTAATC GCCTTGCAGC ACATCCCCCT TTCGCCAGCT GGCGTAATAG CGAAGAGGCC CGCACCGATC GCCCTTCCCA ACAGTTGCGC
7401 AGCCTGAATG GCGAATGGGA CGCGCCCTGT AGCGGCGCAT TAAGCGCGGC GGGTGTGGTG GTTACGCGCA GCGTGACCGC TACACTTGCC AGCGCCCTAG
7501 CGCCCGCTCC TTTCGCTTTC TTCCCTTCCT TTCTCGCCAC GTTCGCCGGC TTTCCCCGTC AAGCTCTAAA TCGGGGGCTC CCTTTAGGGT TCCGATTTAG
7601 TGCTTTACGG CACCTCGACC CCAAAAAACT TGATTAGGGT GATGGTTCAC GTAGTGGGCC ATCGCCCTGA TAGACGGTTT TTCGCCCTTT GACGTTGGAG
7701 TCCACGTTCT TTAATAGTGG ACTCTTGTTC CAAACTGGAA CAACACTCAA CCCTATCTCG GTCTATTCTT TGATTTATA AGGGATTTTG CCGATTTCGG

```
7801 CCTATTGGTT AAAAAATGAG CTGATTTAAC AAAAATTTAA CGCGAATTTT AACAAAATAT TAACGCTTAC AATTTAGGTG GCACTTTTCG GGGAAATGTG
7901 CGCGGAACCC CTATTTGTTT ATTTTTCTAA ATACATTCAA ATATGTATCC GCTCATGAGA CAATAACCCT GATAAATGCT TCAATAATAT TGAAAAAGGA
8001 AGAGTATGAG TATTCAACAT TTCCGTGTCG CCCTTATTCC CTTTTTTGCG GCATTTTGCC TTCCTGTTTT TGCTCACCCA GAAACGCTGG TGAAAGTAAA
8101 AGATGCTGAA GATCAGTTGG GTGCACGAGT GGGTTACATC GAACTGGATC TCAACAGCGG TAAGATCCTT GAGAGTTTTC GCCCCGAAGA ACGTTTTCCA
8201 ATGATGAGCA CTTTTAAAGT TCTGCTATGT GGCGCGGTAT TATCCCGTAT TGACGCCGGG CAAGAGCAAC TCGGTCGCCG CATACACTAT TCTCAGAATG
8301 ACTTGGTTGA GTACTCACCA GTCACAGAAA AGCATCTTAC GGATGGCATG ACAGTAAGAG AATTATGCAG TGCTGCCATA ACCATGAGTG ATAACACTGC
8401 GGCCAACTTA CTTCTGACAA CGATCGGAGG ACCGAAGGAG CTAACCGCTT TTTTGCACAA CATGGGGGAT CATGTAACTC GCCTTGATCG TTGGGAACCG
8501 GAGCTGAATG AAGCCATACC AAACGACGAG CGTGACACCA CGATGCCTGT AGCAATGGCA ACAACGTTGC GCAAACTATT AACTGGCGAA CTACTTACTC
8601 TAGCTTCCCG GCAACAATTA ATAGACTGGA TGGAGGCGGA TAAAGTTGCA GGACCACTTC TGCGCTCGGC CCTTCCGGCT GGCTGGTTTA TTGCTGATAA
8701 ATCTGGAGCC GGTGAGCGTG GGTCTCGCGG TATCATTGCA GCACTGGGGC CAGATGGTAA GCCCTCCCGT ATCGTAGTTA TCTACACGAC GGGGAGTCAG
8801 GCAACTATGG ATGAACGAAA TAGACAGATC GCTGAGATAG GTGCCTCACT GATTAAGCAT TGGTAACTGT CAGACCAAGT TTACTCATAT ATACTTTAGA
8901 TTGATTTAAA ACTTCATTTT TAATTTAAAA GGATCTAGGT GAAGATCCTT TTTGATAATC TCATGACCAA AATCCCTTAA CGTGAGTTTT CGTTCCACTG
9001 AGCGTCAGAC CCCGTAGAAA AGATCAAAGG ATCTTCTTGA GATCCTTTTT TTCTGCGCGT AATCTGCTGC TTGCAAACAA AAAAACCACC GCTACCAGCG
9101 GTGGTTTGTT TGCCGGATCA AGAGCTACCA ACTCTTTTTC CGAAGGTAAC TGGCTTCAGC AGAGCGCAGA TACCAAATAC TGTTCTTCTA GTGTAGCCGT
9201 AGTTAGGCCA CCACTTCAAG AACTCTGTAG CACCGCCTAC ATACCTCGCT CTGCTAATCC TGTTACCAGT GGCTGCTGCC AGTGGCGATA AGTCGTGTCT
9301 TACCGGGTTG GACTCAAGAC GATAGTTACC GGATAAGGCG CAGCGGTCGG GCTGAACGGG GGGTTCGTGC ACACAGCCCA GCTTGGAGCG AACGACCTAC
9401 ACCGAACTGA GATACCTACA GCGTGAGCTA TGAGAAAGCG CCACGCTTCC CGAAGAGAGA AAGGCGGACA GGTATCCGGT AAGCGGCAGG GTCGGAACAG
9501 GAGAGCGCAC GAGGGAGCTT CCAGGGGGAA ACGCCTGGTA TCTTTATAGT CCTGTCGGGT TTCGCCACCT CTGACTTGAG CGTCGATTTT TGTGATGCTC
9601 GTCAGGGGGG CGGAGCCTAT GGAAAAACGC CAGCAACGCG GCCTTTTTAC GGTTCCTGGC CTTTTGCTGG CCTTTTGCTC ACATGTTCTT TCCTGCGTTA
9701 TCCCCTGATT CTGTGGATAA CCGTATTACC GCCTTTGAGT GAGCTGATAC CGCTCGCCGC AGCCGAACGA CCGAGCGCAG CGAGTCAGTG AGCGAGGAAG
9801 CGGAAGAGCG CCCAATACGC AAACCGCCTC TCCCCGCGCG TTGGCCGATT CATTAATGCA GCTGGCACGA CAGGTTTCCC GACTGGAAAG CGGGCAGTGA
9901 GCGCAACGCA ATTAATGTGA GTTAGCTCAC TCATTAGGCA CCCCAGGCTT TACACTTTAT GCTTCCGGCT CGTATGTTGT GTGGAATTGT GAGCGGATAA
10001 CAATTTCACA CAGGAAACAG CTATGACCAT GATTACGCCA AGCGCGCAAT TAACCCTCAC TAAAGGGAAC AAAAGCTGGA GCTGCAAGCT T
```

酶切鉴定

限制性内切酶	位点	片段大小（bp）
NheI	1798, 3774	1976, 8115
AgeI	2038	10091
ApaLI	3798, 4137, 8122, 9368	339, 3985, 1246, 4521
ApaLI+AgeI	2038, 3798, 4137, 8122, 9368	1760, 339, 3985, 1246, 2761
ApaLI+NheI	1798, 3774, 3798, 4137, 8122, 9368	1976, 24, 339, 3985, 1246, 2521
