# Supplementary figures and images for: Macrophages release IL11-containing filopodial tip vesicles and contribute to renal interstitial inflammation
Source: Cell Commun Signal. 2023 Oct 18;21:293. doi: 10.1186/s12964-023-01327-6 (PMC10585809; doi:10.1186/s12964-023-01327-6)

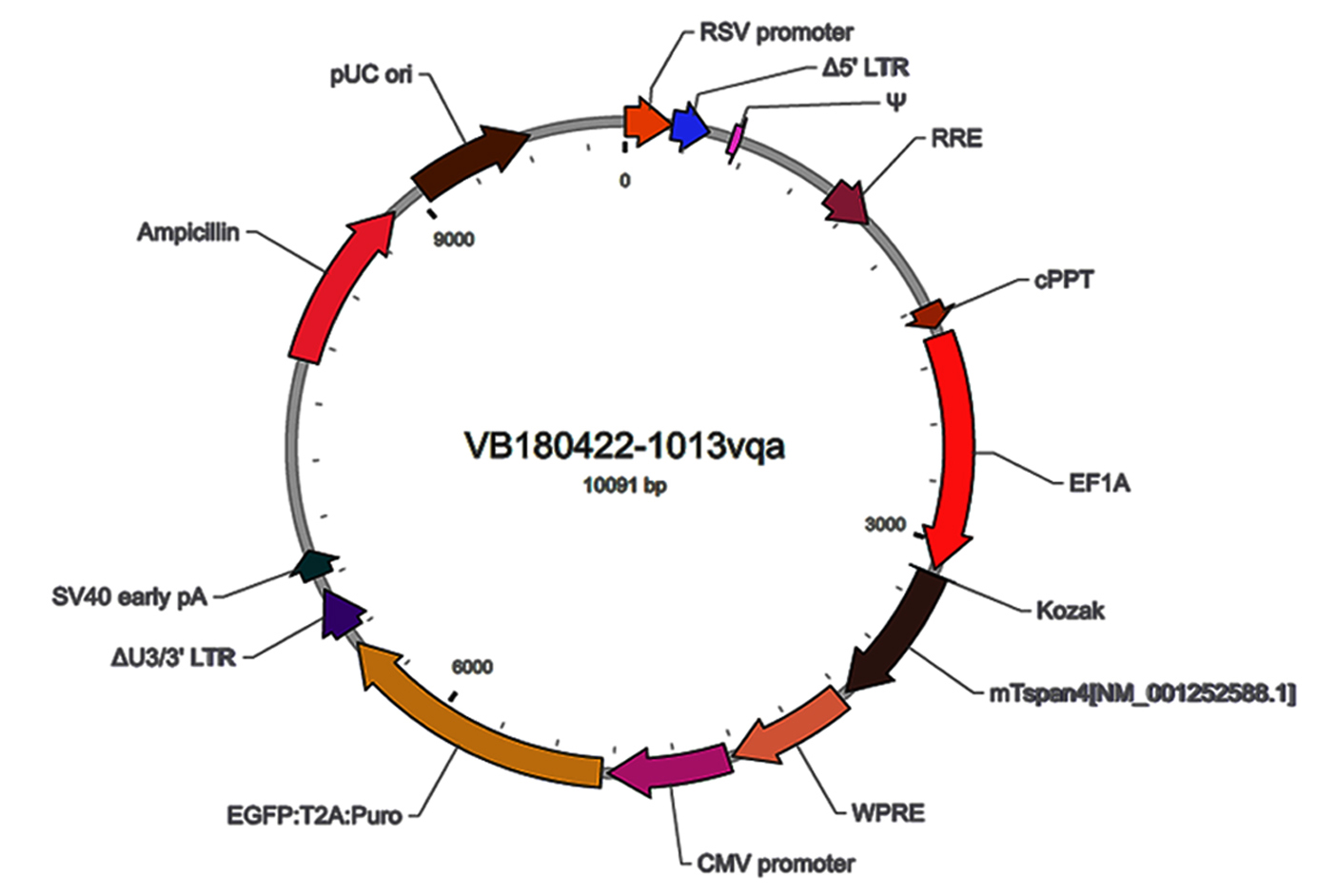

Supplement: Supplementary file 5 — Additional file 4. [file 12964_2023_1327_MOESM4_ESM.jpg]

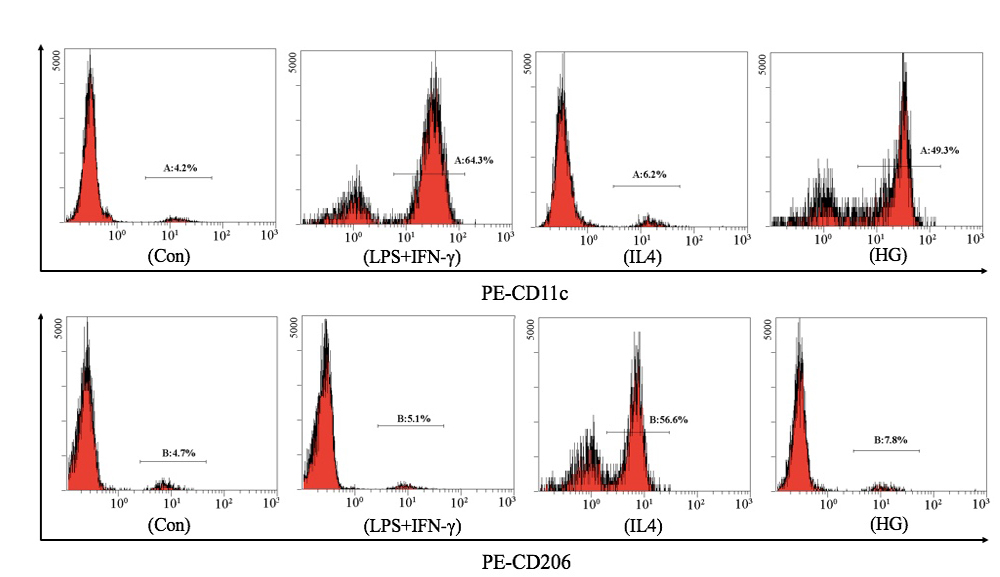

Supplement: Supplementary file 6 — Additional file 5. [file 12964_2023_1327_MOESM5_ESM.jpg]

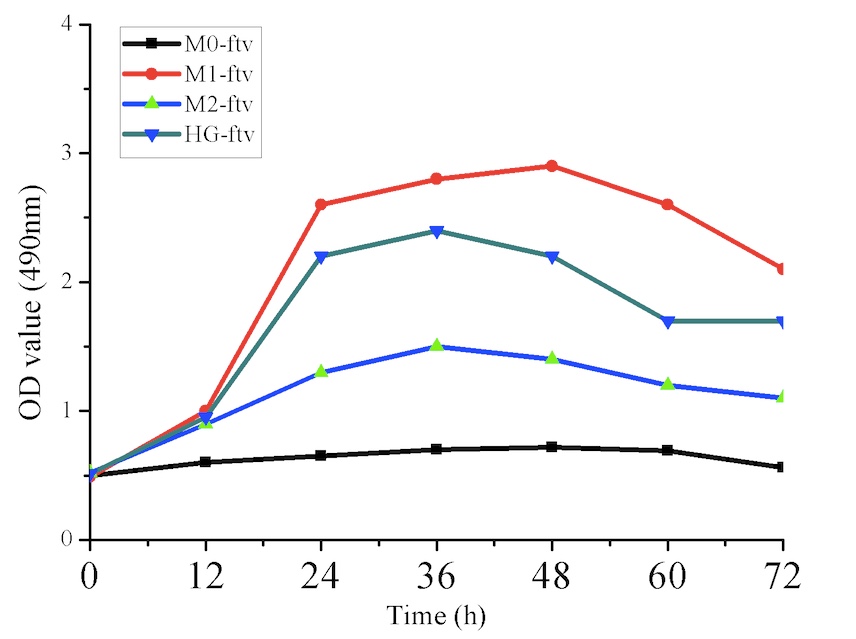

Supplement: Supplementary file 7 — Additional file 6. [file 12964_2023_1327_MOESM6_ESM.jpeg]
